# Eukaryotic Voltage-Gated Sodium Channels: On Their Origins, Asymmetries, Losses, Diversification and Adaptations

**DOI:** 10.3389/fphys.2018.01406

**Published:** 2018-11-21

**Authors:** Julia E. Fux, Amrit Mehta, Jack Moffat, J. David Spafford

**Affiliations:** Department of Biology, University of Waterloo, Waterloo, ON, Canada

**Keywords:** sodium channels, calcium channels, auxiliary beta subunits, evolution, ion selectivity, NALCN, patch clamp electrophysiology, U12-type splice site

## Abstract

The appearance of voltage-gated, sodium-selective channels with rapid gating kinetics was a limiting factor in the evolution of nervous systems. Two rounds of domain duplications generated a common 24 transmembrane segment (4 × 6 TM) template that is shared amongst voltage-gated sodium (Na_v_1 and Na_v_2) and calcium channels (Ca_v_1, Ca_v_2, and Ca_v_3) and leak channel (NALCN) plus homologs from yeast, different single-cell protists (heterokont and unikont) and algae (green and brown). A shared architecture in 4 × 6 TM channels include an asymmetrical arrangement of extended extracellular L5/L6 turrets containing a 4-0-2-2 pattern of cysteines, glycosylated residues, a universally short III-IV cytoplasmic linker and often a recognizable, C-terminal PDZ binding motif. Six intron splice junctions are conserved in the first domain, including a rare U12-type of the minor spliceosome provides support for a shared heritage for sodium and calcium channels, and a separate lineage for NALCN. The asymmetrically arranged pores of 4x6 TM channels allows for a changeable ion selectivity by means of a single lysine residue change in the high field strength site of the ion selectivity filter in Domains II or III. Multicellularity and the appearance of systems was an impetus for Na_v_1 channels to adapt to sodium ion selectivity and fast ion gating. A non-selective, and slowly gating Na_v_2 channel homolog in single cell eukaryotes, predate the diversification of Na_v_1 channels from a basal homolog in a common ancestor to extant cnidarians to the nine vertebrate Na_v_1.x channel genes plus Nax. A close kinship between Na_v_2 and Na_v_1 homologs is evident in the sharing of most (twenty) intron splice junctions. Different metazoan groups have lost their Na_v_1 channel genes altogether, while vertebrates rapidly expanded their gene numbers. The expansion in vertebrate Na_v_1 channel genes fills unique functional niches and generates overlapping properties contributing to redundancies. Specific nervous system adaptations include cytoplasmic linkers with phosphorylation sites and tethered elements to protein assemblies in First Initial Segments and nodes of Ranvier. Analogous accessory beta subunit appeared alongside Na_v_1 channels within different animal sub-phyla. Na_v_1 channels contribute to pace-making as *persistent* or *resurgent* currents, the former which is widespread across animals, while the latter is a likely vertebrate adaptation.

## Introduction to the Superfamily of 4 × 6 Tm Voltage-Gated Cation Channels

**FIGURE 1:**
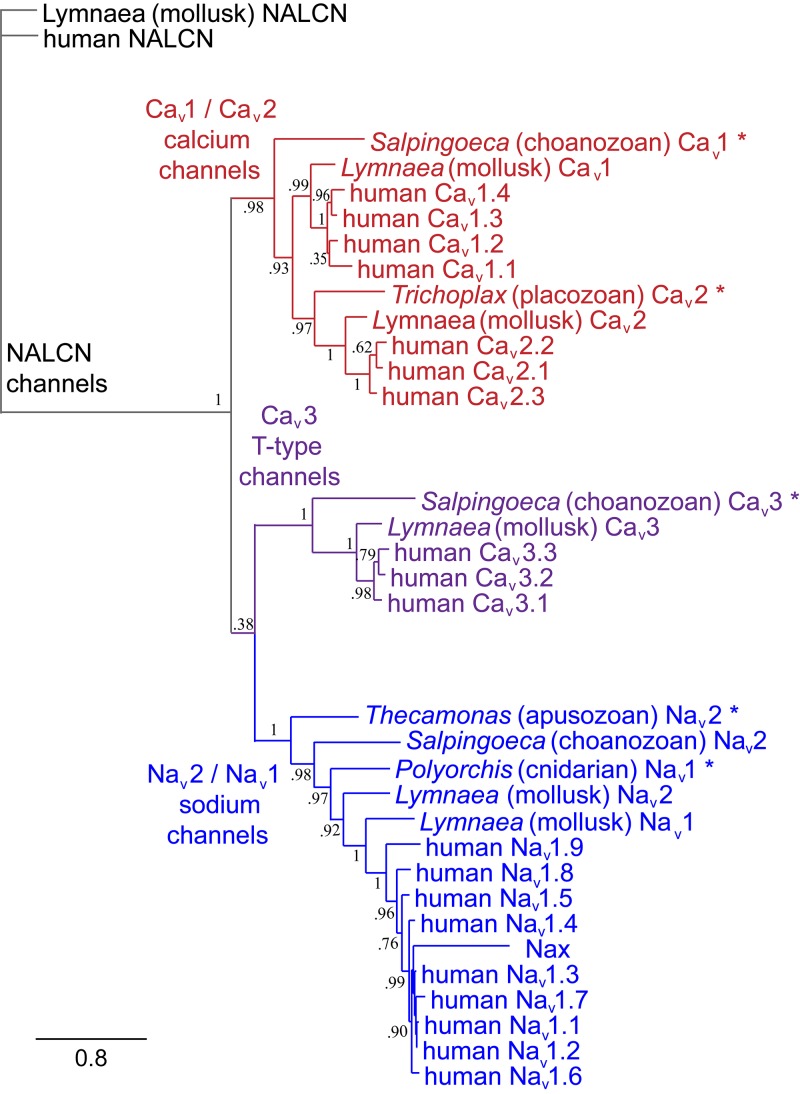
Gene tree of 4 × 6 TM (TM = transmembrane) voltage-gated sodium channel α subunits (Na_v_1 and Na_v_2), calcium channel α_1_ subunits (Ca_v_1, Ca_v_2, and Ca_v_3) and NALCN. Included in the multiple alignment are the 21 human homologs and the 6 homologs from representative protostome invertebrate, pulmonate snail, *Lymnaea stagnalis*. Basal genes of each class (indicated in bold and ^∗^) including apusozoan, *Thecamonas trahens* (Na_v_2), single cell choanoflagellate, *Salpingoeca rosetta* (Ca_v_1 and Ca_v_3), placozoan, *Trichoplax adhaerens* (Ca_v_2), and cnidarians (hydrozoan jellyfish), *Polyorchis penicillatus* (Na_v_1). More distant Na Leak Conductance channel (NALCN) serve as the out-group for the phylogenetic tree. Bootstrap values are shown at the nodes. Notably weak branch strength is in the positioning for Ca_v_3 T-type channels which is relatively equidistant from sodium channels and other calcium channels. The gene tree illustrate the close kinship amongst Na_v_2 and Na_v_1 channels and between Ca_v_1 and Ca_v_2 channels. Amino acid sequences were aligned using MUSCLE 3.7 (Edgar, 2004) within EMBL-EBI web interface (Chojnacki et al., 2017). Gene trees were constructed in PhyML 3.0 (Guindon et al., 2010) using Phylogeny.fr web interface (Dereeper et al., 2008).

The superfamily of voltage-gated cation channels containing 24 transmembrane segments are classified as sodium-selective, calcium-selective, or non-selective channels that span the plasma membrane. Their gene numbers, their expression patterns and functions have been reported in differing body plans that range from single cell eukaryotes, to invertebrates, to the greatest gene complexity of ion channel isoforms in vertebrates. There are twenty-one mammalian genes : Ten are sodium channel (SCNxA) genes coding for Na_v_1.1 to Na_v_1.9 (Catterall et al., 2005a), expressing mostly sodium-selective currents and Nax (Noda and Hiyama, 2015); Ten are calcium channel (CACNA1x) genes, coding for Ca_v_1.1 to Ca_v_1.4, Ca_v_2.1 to Ca_v_2.3, and Ca_v_3.1 to Ca_v_3.3 (Catterall et al., 2005b), that generate mostly calcium-selective channel currents (Figure 1). NALCN is a unique, orphan, leak channel gene within the superfamily of voltage-gated cation channels (Cochet-Bissuel et al., 2014; Figure 1) but are poorly understood, because its ion channel characteristics have not been identified and validated by *in vitro* expression (Senatore et al., 2013; Senatore and Spafford, 2013; Boone et al., 2014). Here, we start by exploring the features in key extant life forms which provide insights into the evolutionary history of the voltage-gated, sodium-selective channels. The most basal life form for appearance of voltage-gated sodium channel classes is in the single cell eukaryotes (Zakon, 2012). Prokaryotes have representative ion channels for potassium- (Doyle et al., 1998) and sodium-selective (Yue et al., 2002) pores, which provides insights into the selectivity mechanisms of eukaryotic 4 × 6 TM channels.

## Potassium-Selective, Voltage-Gated Channels Share a Common Pore Structure in Bacteria and Eukaryotes

**FIGURE 2:**
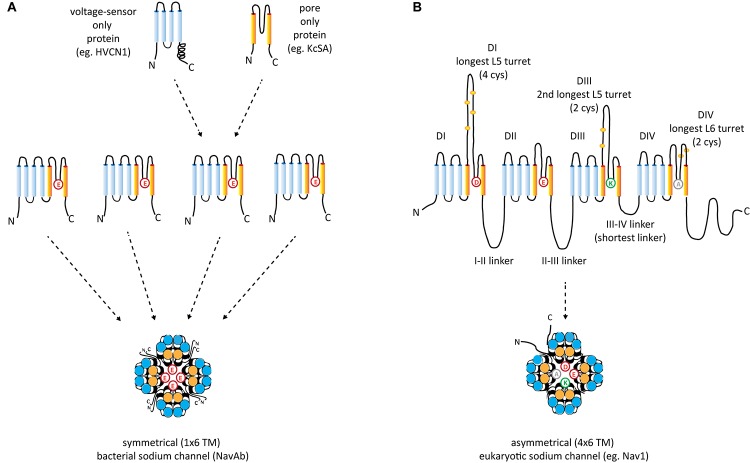
Structural comparisons between **(A)** symmetrical, 1 × 6 TM (TM = transmembrane) homotetrameric, bacterial Na channels consisting of four repeat subunit **(B)** asymmetrical, eukaryotic four domain calcium and sodium channels consisting of 24 transmembrane segments (4 × 6 TM). Each of the four sodium and calcium channel domains consist of a voltage-sensor domain of four transmembrane segments, S1–S4 (blue), akin to voltage sensor only protein (e.g., HVCN1) and a pore domain, S5–S6 (orange), resembling the size of the two membrane segments of the inward rectifying K channels (e.g., KcsA). A dramatic difference between the 1 × 6 TM bacterial sodium channels and 4 × 6 TM calcium and sodium channels are the larger sizes of extracellular turrets rising before the pore selectivity filter, especially L5_I_ and L5_III_ which range from 40 to 105 amino acids long. The longest extracellular turret in Domain IV in 4 × 6 TM channels is always the extracellular turret rising after the pore selectivity filter (L6_IV_), while L5_IV_ (the extracellular loop just before the pore selectivity filter) is always short. Comparatively speaking, 1 × 6 TM channels all possess short extracellular turrets of 10–15 amino acids long. Eight core, conserved cysteines populate the L5_I_ - L5_II_ - L5_III_ - L6_IV_ extracellular turrets in a 4-0-2-2 configuration in all eukaryotic 4 × 6 TM channels. Also novel are singleton N- and C-termini, and cytoplasmic linkers, of which the III-IV linker is almost always 53–57 amino acids long in eukaryotic 4 × 6 TM channels.

**FIGURE 3:**
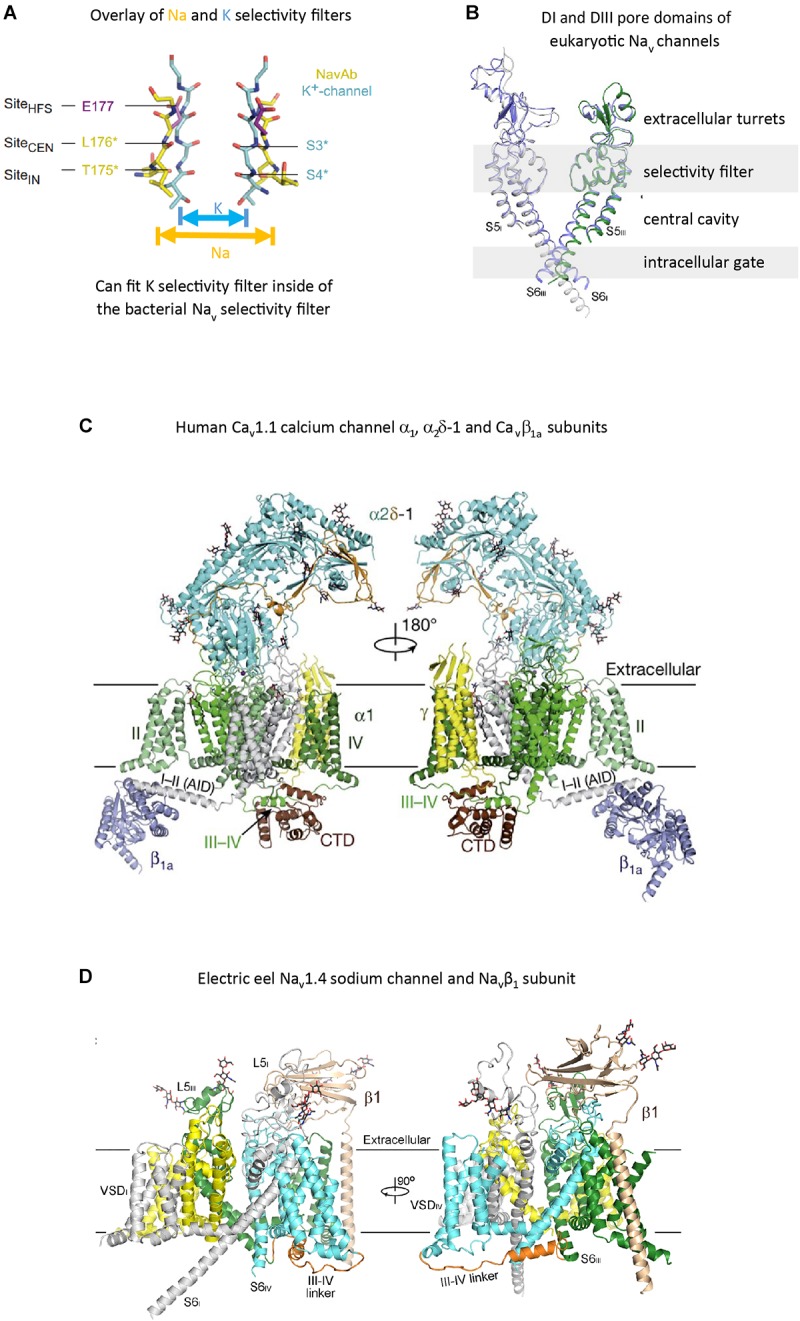
High resolution structures of pore selectivity filters, a pore domain and full length voltage gated calcium and sodium channels. **(A)** Overlay of the structure of the pore selectivity filter of bacterial KcsA potassium channel (Doyle et al., 1998) and bacterial sodium channel Na_v_Ab (Payandeh et al., 2012). Only two subunits are shown for clarity. Backbone carbonyls in the selectivity filter are red. The narrower pore selectivity filter for potassium ions fits within the broader selectivity filter for sodium (or calcium) ions. **(B)** Overlay of Domain I and III pores of arthropod Na_v_1 and electric eel Na_v_1.4. Key amino acid side chain residues of the selectivity filter project into the pore center to regulate ion passage through sodium and calcium channels pores. Long extensive extracellular turrets rising from segment 5 to the pore selectivity filter, and shorter extensions from the pore selectivity filter to segment 6 project rise above the membrane, serving as a first contact for incoming ions and drugs, before passing through the selectivity filter below. **(C)** α1 subunit of the human Ca_v_1.1 calcium channel, illustrating the extended extracellular turret loops of L5_I_, L5_III_, L6_IV_ and the intracellular globular domain formed by the III-IV linker and the proximal C-terminal Domain (CTD) containing the calmodulin binding IQ motif. The proximal C-terminus omitted in the right panel to better illustrate the III–IV linker. Accessory α2Δ-1 and β1A subunits are illustrated with the pore-forming α1 subunit. Potential Ca^2+^ ions in the selectivity filter vestibule are illustrated as green spheres. **(D)** The structure of the electric eel Na_v_1.4 (each domain individually colored) and β1(wheat colored) complex, with glycosyl moieties shown as black sticks. The short cytoplasmic III-IV linker (orange color) regulates fast inactivation of Na_v_1 channels. **(A)** reproduced from Payandeh, et al (2011). *Nature*, 10:475(7356):353-8 with permission. **(B,D)** reproduced from Yan et al. (2017). **(C)** reproduced from J Wu et al. (2016).

The voltage-gated (K^+^, Ca^2+^, and Na^+^) channels consist of two semi-autonomous domains, a pore domain that provides an aqueous pathway for ion selection through the plasma membrane, and a voltage-sensor domain, that transduces pore gate movements (opening/closing events), based on changes to the state of the membrane electric field. There are two major templates for the pore domain that first appear in bacterial representatives, one that is a highly selective pore domain for potassium ions (Doyle et al., 1998) and a different pore domain that is selective for sodium ions (Payandeh et al., 2011). The overall structure of pore domains consist of two transmembrane helices (the so-called S5–S6 segments in voltage-gated channels), with an intervening Pore- (P-) loop, that forms a pore lining border that resembles an inverted teepee, including a descending pore helix from the end of S5 and an ascending loop to the end of S6 (Kim and Nimigean, 2016; Figure 2). At the base of the inverted teepee between the pore helices is a pore-selectivity filter that ascends to form the most constricted point of access of ions, between the outer vestibule facing the wide, extracellular milieu above, and the more expansive aqueous lake within the membrane channel below (Kim and Nimigean, 2016) (Figures 3A,B). The first of the high resolution structures of the potassium selective pore is KcSA, a pore only protein isolated from the bacteria, *Streptomyces lividans* (Doyle et al., 1998). The signature sequence of the potassium selective pore is the five amino acid residues: T(V/I)GYG contributed by each of four subunits forming identical quadrants contributing to the pore lining selectivity filter. The side chains residues face outwards, and the pore-lining backbone carbonyls form an octet of oxygens, serving to accommodate four dehydrated potassium ions, occupied in every other position (1 and 3 or 2 and 4) at one time in potassium channels (Doyle et al., 1998; Figure 3A). The K channel pore achieves an exclusive K ion selectivity because only the K channel will shed its hydration shell, for optimized, energetically favorable binding conditions within the pore selectivity filter, and passage through by electrochemical force generated by a transmembranal ion gradient (Doyle et al., 1998). Both prokaryotic and eukaryotic potassium-selective channels share the same signature, selectivity filter of T(V/I)GYG residues (Kim and Nimigean, 2016).

## The Four Domains of Eukaryotic Ca_v_ and Na_v_ Channels has Resemblances With the Single Domain of the Bacterial Na_v_ Channel

The overall structure for the sodium-selective pore in eukaryotes approximates the unique dimensions of a sodium-selective channel pore identified in bacteria, exemplified by Na_v_Ab, the first bacterial sodium channel resolved by X-ray crystallography, isolated from *Arcobacter butzleri* (Payandeh et al., 2011). The pore selectivity filter is a broader and shorter conduit for ion passage across the membrane, such that one can fit the K ion-selective pore between the dimensions of the bacterial or eukaryotic Na -selective pore (Payandeh et al., 2011; Figures 3A,B). There is an additional pore helix ascending from the ion selectivity filter (P2 helix) in both bacteria sodium channels (Payandeh et al., 2011) and eukaryotic sodium and calcium channels (Wu et al., 2016), in addition to the descending pore helix leading to the ion selectivity filter (P1 helix, also shared in potassium channels), generating a wider and more structured outer vestibule facing the channel exterior.

The equivalent of the potassium channel’s signature residues of the pore selectivity filter is TL**E**SWSM in the bacteria sodium channel (Figure 3A), where the negatively charged carboxylates side chain of the glutamate residue (**E**) faces towards the pore center (rather than away from the pore center of the K selective pore), creating a highly electronegative, high field strength (HFS) site, contributed by the four glutamates (EEEE) in identical position (Payandeh et al., 2011). At this most constricted width of the Na ion selective pore, is a 4.6 × 4.6 angstrom square HFS site, that is large enough to accommodate a sodium ion with two planar waters of hydration (Payandeh et al., 2011; Figure 3A). This contrasts with the narrow ion traversing pathway where all waters are stripped from the potassium ion before entering the pore selectivity filter (Figure 3A).

Potassium-selective pores can be voltage-gated with addition of N-terminally attached voltage-sensor domains, consisting of four (S1–S4) segments (Groome, 2014). The voltage-sensor is optional in potassium channels, and can operate as a semi-autonomous unit. An example of voltage-sensor only proteins are the voltage-gated proton channels that allow proton transport into phagosomes via the voltage sensor (e.g., human HVCN1) (Takeshita et al., 2014; Figure 2A). All eukaryotic sodium and calcium channels possess voltage-sensor domains like the voltage-gated potassium channel, where the S4 segments have a variable number (4–8) positive charged residues (lysine or arginine) every third amino acid, forming a highly charged side of an alpha helix (Groome, 2014). S4 segments respond to membrane potential changes, by movement of their positive charged residues along negatively charged, counter-charges formed by S1–S3 segments, transducing the mechanical coupling of an amphipathic S4–S5 helix to pore gating movements that lead to iris-like, occlusion or widening of the pore helical bundle formed by the distal ends of S6 segments (Groome, 2014).

## Tracing of Domain Duplications to Generate the Four Domain Ca_v_ and Na_v_ Channels From a Single Domain Ancestor

Sodium and calcium channels only exist in as voltage-gated channels subunits with voltage-sensors and pore domains (6TM, TM = TransMembrane) fused together, unlike potassium channels which can exist as pore-only proteins (Figure 2A). The 1 × 6 TM bacterial sodium channels have been identified in proteobacteria and actinobacteria, but also have spread to eukaryotes, including diatoms, which they likely have received by horizontal gene transfer from bacteria (Verret et al., 2010).

**FIGURE 4:**
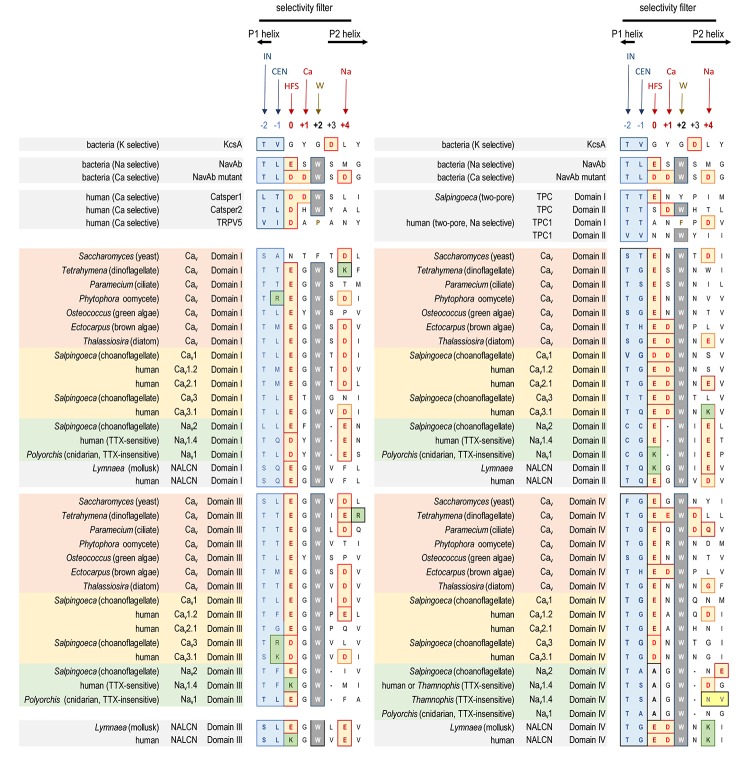
Alignment of amino acid sequences contributing to the pore selectivity filters in representative calcium, sodium and NALCN channel homologs from single cell protists, yeast, algae, invertebrate and human, as well as 2 × 6 TM TPC channels, 1 × 6 TM channels (TRP, Catsper, and bacterial Na), and 1 × 2 TM channels (bacterial K). The pores of 4 × 6 TM channels from brown and green algae, yeast, single-cell choanoflagellates, cnidarian and mollusks are illustrated alongside human representatives, to illustrate the relationship of pore selectivity filters in different life forms. The selectivity filter is flanked by a descending P1 helix and ascending P2 helix. Residues contributing to the central (CEN) and inner (IN) sites of the selectivity filter are highlighted blue. Negative charged residues contributing to the selectivity filters (HFS sites), and to the ring of outer carboxylates are a red/brown color. A positively charged lysine residue (green color) populate the HFS site in Domain II or III of all Na_v_1 channels. The aspartate (D) residue in Domain II at the Ca site, next to the HFS site, is conserved in Ca_v_1, Ca_v_2 and Ca_v_3 channels. Na_v_2 and Na_v_1 channels are shortened in the pore selectivity filter in Domain II and lack the Ca site altogether. Instead, a glutamate (E) residue is conserved at the Na site (HFS+4) in Domain II of Na_v_2 and Na_v_1 channels. Exceptionally conserved tryptophans (W site) form inter-repeat hydrogen bonds that stabilize the pore loop region. Outer ring carboxylates (Na site, especially in Domain IV) contribute to TTX sensitivity, which when altered lowers TTX insensitivity. Note the amino acid changes (yellow colored residues) in Domain IV of the TTX-sensitive hNa_v_1.4 and TTX-low sensitive channels from garter snake (*Thamnophis sirtalis*) that adapts to feed on TTX-ladened newts by neutralizing a negative charge at the Na site. Bacterial Na channel, Na_v_Ab becomes calcium-selective with aspartate (D) substitutions at the HFS, Ca and Na sites. Amino acid sequences were aligned using MUSCLE 3.7 (Edgar, 2004) within EMBL-EBI web interface (Chojnacki et al., 2017).

Typical eukaryotic calcium and sodium channels are always approximately four times the equivalency in size of 1 × 6 TM prokaryotic sodium channels and voltage-gated potassium channels (Figure 2B). The closest homolog to the prokaryotic 1 × 6 TM Na-selective channels are the eukaryotic transient receptor potential (TRP) family of channels, whose members are usually non-selective or sodium selective channels, with exceptions, such as Catsper1 with an electronegative HFS sites which are key for conferring a calcium-selectivity (Wu et al., 2010; Figure 4). Two domain TPC channels are sodium-selective in mammals, but TPC homologs in basal representatives such as *Salpingoeca rosetta* resemble calcium-selective channels with a glutamate or aspartate in Domains I or II of the HFS site of the pore selectivity filter (Figure 4). The example of differing ion selectivities in TPC channels is suggestive of experimentation between sodium and calcium selective pores in possible two domain channel ancestors to eukaryotic 4 × 6 TM channels (Peng et al., 2015). As expected from a two domain intermediate that duplicated into a four domain channel such as 4 × 6 TM channels (Strong et al., 1993), the first domain of TPC is more similar to Domain I and III of 4 × 6 TM channels and the second domain of TPC is more similar to Domain II and IV of 4 × 6 TM channels (Rahman et al., 2014).

## Evidence in Genomic Structure for a Common Calcium and Sodium Channel Template

**FIGURE 5:**
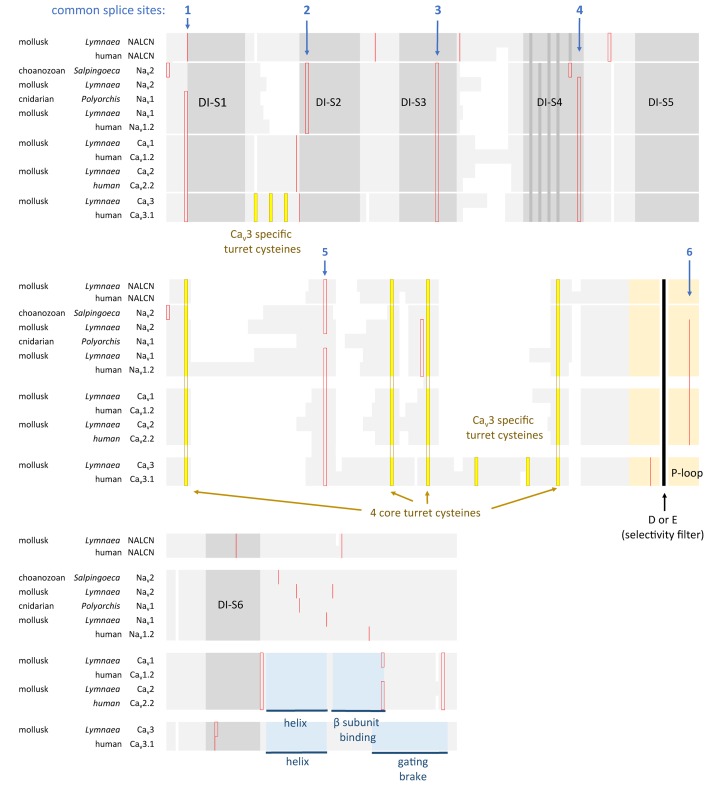
Amino acid alignment of the first domain of representative 4 × 6 TM channels illustrating the conservation of six intron splice sites (red vertical lines) in voltage-gated calcium (Ca_v_1, Ca_v_2, and Ca_v_3) and sodium (Na_v_1 and Na_v_2) channels. Conserved placement of intron splice junctions (red vertical lines) span each transmembrane segment of the voltage-sensor domain (D1S1, D1S2, D1S3, and D1S4) and two in the pore loop of Domain I (DIS5-D1S6). The sixth splice site is lacking in Ca_v_3 channels. NALCN lacks all of these splice sites, supporting a more distant relationship for NALCN compared to the eukaryotic calcium and sodium channels. The six membrane spanning helices S1–S6 are indicated in gray shading, with the High Field Strength (HFS) site (D or E) indicated by black vertical line surrounded by the rest of the pore loop region (light orange shading). Darker gray shading in S4 indicate the positions of positive charges serving the voltage-sensor domain. Four cysteine residues are conserved in the extracellular turret (L5) of all 4 × 6TM channels, and the location of two extra cysteines in L5 and S1–S2 are only found in Ca_v_3 T-type channels. Cysteine residues are indicated in a bright yellow color. Illustrated in blue color is a rigid, helix in the proximal I-II cytoplasmic linker, upstream of an accessory Ca_V_β subunit binding site in Ca_v_1 and Ca_v_2 channels, and the “gating brake” of Ca_v_3 channels which harbors a nano-molar affinity binding site for calmodulin. Genes in the alignment include NALCN, sodium channels (Na_v_2 and Na_v_1) and calcium channels (Ca_v_1, Ca_v_2, and Ca_v_3) from human representatives, as well as protostome invertebrate, pond snail *L. stagnalis*, and basally branching species from single cell choanoflagellate, *S. rosetta*, placozoan, *Trichoplax adhaerens*, and cnidarians: *Nematostella vectensis* (sea-anemone) and *Polyorchis penicillatus* (hydrozoan jellyfish). Amino acid sequences were aligned using MUSCLE 3.7 (Edgar, 2004) within EMBL-EBI web interface (Chojnacki et al., 2017).

**FIGURE 6:**
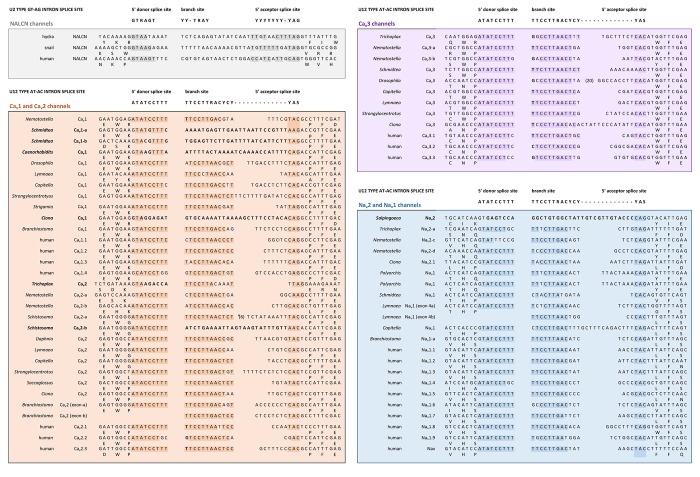
Conservation of a very rare U12 type intron splice site in Domain 1, segment 1 of Na_v_2, Na_v_1, Ca_v_1, Ca_v_2, and Ca_v_3 channels, but not NALCN channels. Exceptionally rare U12-type splice site represent just 700–800 putative genes (or ∼4%) of total genes in mammalian genomes. Consensus 5′ donor splice site (ATATCCTTT), branch site (TTCCTTRACYCY) and 5′ acceptor splice site (YAS) recognition sequences of minor U12-type spliceosome varies from conventional major U2-type spliceosome recognition sequences with consensus 5′ donor splice site (GTRAGT), branch site (YY-TRAY) and 5′ acceptor splice site (YYYYYYY-YAG). NALCN has a variable intron splice junction in this region, with resemblances to the major U2-type spliceosome recognition sequence. DNA sequences were aligned using MUSCLE 3.7 (Edgar, 2004) within EMBL-EBI web interface (Chojnacki et al., 2017).

All the 4 × 6 TM channels of the superfamily of Ca_v_ and Na_v_ channels appear to derive from the same stem eukaryotic channel with a fundamentally shared template. There is evidence in a shared genomic structure: Sodium channels (Na_v_2 and Na_v_1) and calcium channels (Ca_v_1, Ca_v_2, and Ca_v_3) in animals possess five common splice site locations in the first of four domains, including each of the first four transmembrane segments (S1–S4) of Domain I of the voltage-sensor domain (D1S1, D1S2, D1S3, and D1S4) and two splice site locations in the pore loop (S5–S6) of Domain I (DIS5-D1S6) (Figure 5). The D1S1 splice site is conserved in almost all known calcium and sodium channels (Spafford et al., 1999) and is a highly rare, unconventional, U12- type (AT-AC) splice site that representing just 700-800 putative genes (or ∼4%) of total genes in mammalian genomes (Parada et al., 2014; Figure 6). The exception in the conservation of splice sites in Domain I is that the 6th splice site in the D1 pore loop is lacking in Ca_v_3 channels (Figure 5). A shared genomic structure in the first of four domain may indicate constraints on the structural divergence of the first domain shared between four domain calcium and sodium channels. These six conserved splice sites are not present in NALCN channels (Figure 5), including the unconventional, U12- type splice site governed by the minor spliceosome shared in homologous position of D1S1 (Figure 5). The lack of conservation of intron splice sites reflects the weaker evolutionary link between NALCN from the cluster of more closely related families of eukaryotic voltage-gated calcium and sodium channels.

## A Common Asymmetrical 4 × 6 Tm Channel Structure is Shared Amongst Most Groups of Eukaryotes

**FIGURE 7:**
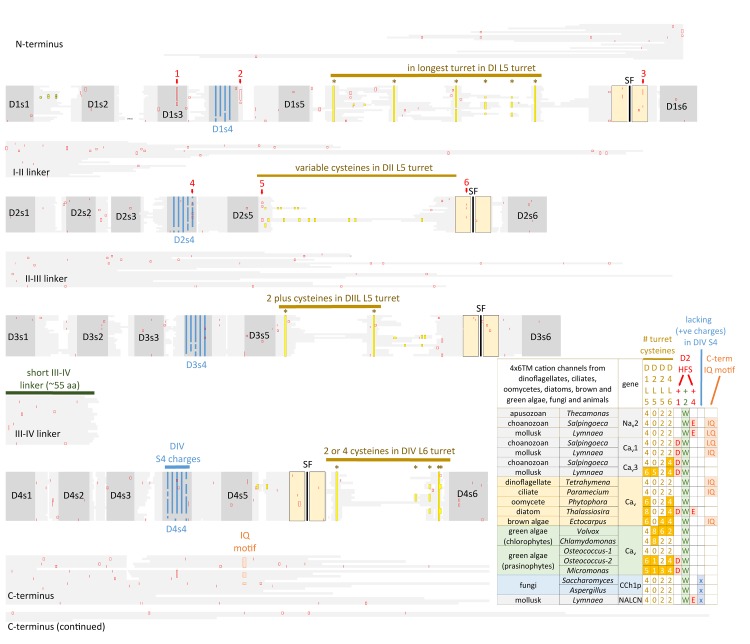
Alignment of 4 × 6 TM cation channel representatives illustrating a shared fundamental architecture of asymmetrically arranged extracellular and intracellular regions from single cell protists (choanoflagellates, dinoflagellates, oomycetes, and ciliates), green algae (chlorophytes and prasinophytes), brown algae, yeast, and Na_v_2, Na_v_1, Ca_v_1, Ca_v_2, and Ca_v_3 channels from protostome invertebrate (pond snail, *L. stagnalis*). A concerted evolutionary change to a common asymmetrical architecture of 4 × 6 TM cation channels contrasts with the pseudo-symmetry of cyclic nucleotide-gated potassium channel (CNGK) channels which contain four nearly identical domains chained together. Long extensive extracellular turrets prior to the pore selectivity filter (SF) include L5_1_, L5_III_ and more rarely L5_II_ and a longer turret after the SF in L6_IV_, as well as a 4-0-2-2 framework of core cysteines populating L5_I_-L5_II_-L5_III_-L6_IV_ extracellular turrets in 4 × 6 TM channels. L5_IV_ extracellular turret is always short in 4 × 6 TM channels. The cytoplasmic III-IV linkers are always short (∼53–57 amino acids) compared in contrast to more variable I-II linker, II-III linkers, and N- and C-termini in 4 × 6 TM cation channels. Intron splice sites are indicated by red vertical lines. Dinoflagellate, ciliate, oomycete, diatom, green and brown algae channels illustrated in the figure all possess a conserved HFS site resembling an animal calcium channel (EEEE). Notably lacking in most of these protist channels is the conservation in the pore selectivity filter of Domain II of a “*calcium beacon*” aspartate (D) (HFS+1) omni-present in animal calcium channels, or a “*sodium beacon*” glutamate (HFS+4) omni-present in animal sodium channels, and a C-terminal IQ motif. A tryptophan ring at HFS+2 is present in all four domains of all channels except Domain I of Cch1p. A notable absence of S4 charges is found for yeast Cch1p and NALCN. Amino acid sequences were aligned using MUSCLE 3.7 (Edgar, 2004) within EMBL-EBI web interface (Chojnacki et al., 2017).

Eukaryotic 4 × 6 TM channels adopted a particular asymmetrical, architecture followed domain duplication which generated the four domain channel. The signature, asymmetrical structure is common to all known cation channels including sodium, calcium and NALCN channels in animals, including Na_v_2, Ca_v_1, and Ca_v_3 homologs in the single cell choanoflagellate, *S. rosetta*, voltage-gated cation channels from other protists (e.g., dinoflagellates, ciliates, oomycetes, and diatoms), yeast calcium channel (e.g., Cch1p) (Locke et al., 2000), as well as species of brown and green algae (chlorophytes and prasinophytes) (Figure 7). Major eukaryotic groups lacking the 4 × 6 TM channel structure are the embryophytes (the land plants) and red algae (Verret et al., 2010). Ancestors to the land plants appeared to have purged the eukaryotic 4 × 6 TM channels, alongside key components of the animal toolkit for cation influx across the plasma membrane which includes the IP3 receptors, ATP-gated purinergic receptors, the cys-loop superfamily of ligand-gated ion channels and TRP channels (Edel et al., 2017). A single two-domain, TPC1 homolog is retained in land plants as it relates to comparable signaling across internal membrane compartments of plant vacuoles and animal organelles (Hedrich and Marten, 2011).

## Organization of Pore and Voltage Sensor Domains in four Domain Cation Channels

Generation of the four pores and voltage-sensor domains after domain duplication, provided opportunity for divergence and specialization of the four individual domains of 4 × 6 TM channels. This is evident in studies assessing the relative contribution of voltage-sensor domains in Na_v_1 channels for example, where the first three of four domains appear to be faster in gating charge movement, and are necessary and sufficient to regulate channel opening, while the fourth domain is slower to mobilize and contributes more to the refractory, inactivated state of the channel after prolonged channel opening (Capes et al., 2013).

Associated structural changes for the coordinated movements of individual domains for channel gating include a unique “domain swapping” topology found in 1x6TM (Doyle et al., 1998; Payandeh et al., 2011) and 4 × 6 TM (Wu et al., 2015; Shen et al., 2017) voltage-gated cation channels, where voltage sensor modules are rotated clockwise with respect to pore modules of each domain. The voltage sensor module of Domain I, for instance, surrounds the Domain II pore. The domain swapping arrangement is lacking in many potassium channel classes [e.g., Eag1 (Whicher and MacKinnon, 2016), CNG (Li et al., 2017), HCN (Lee and MacKinnon, 2017) Slo1 (Tao et al., 2017) and Slo2.2 (Hite and MacKinnon, 2017)], and lacking in some TRP channels like TRPV6 while present in other TRP channels (e.g., TRPV1 and TRPV2) (Singh et al., 2017). The absence of a domain swapping topology correlates with an S4-S5 linker that is too short to accommodate the swapping of voltage-sensor and pore domains. The longer S4-S5 linkers supports a rotary coupling of interlocked pore domains for more concerted actions in gated pore movements within voltage-gated cation channels (Bagneris et al., 2015).

## Conservation of Positioning of Cysteines and Patterns of Extracellular Turret Sizes Within Eukaryotic 4 × 6 Tm Channels

There is a specific asymmetrical pattern of long (15–105 aa) extracellular turrets shared in the pore domains of all animal calcium, sodium and NALCN channels, and more broadly with most eukaryotic lifeforms (outside of land plants and red algae), including yeast, ciliates, dinoflagellates, oomycetes, diatoms and brown and green (chorophytes and prasinophytes) algae (Figures 2, 7). Bacterial sodium and potassium channels possess extracellular L5 and L6 turrets that are always short (10–15 aa) in length (Stephens et al., 2015; Figures 2, 7). 4 × 6 TM channels possess a rising extracellular turret from the S5 transmembrane helix (dubbed L5) before the pore selectivity filter, and a second shorter extracellular turret after the pore before S6 transmembrane helix (dubbed L6) (Figures 2, 3C,D, 7; Stephens et al., 2015). The longest L5 extracellular turret is in Domain I (ranging from 60 to 105 aa). L5 in Domain III possess the second longest turret (ranging from 40 to 60 aa), while Domain II and IV turrets are the shortest L5 extracellular turrets (15–30 aa) in size. The longest and most variable L6 extracellular turret amongst 4 × 6 TM channels is contained in Domain IV, with L6 extracellular turrets being shorter and less variable in Domains I, II and III. Populated within the four turret domains is a fundamental set of eight conserved cysteines that are present in all 4x6 TM channels: two in L5_I_, two in L5_III_ and two in L6_IV_ (Figure 7; Stephens et al., 2015). The extended extracellular turrets appear to form intra-loop disulphide bonds which stabilize the structure as a “windowed dome” of interlocked extracellular loops in the human Ca_v_1.1 calcium channel (Figure 3C; Wu et al., 2016) or electric eel Na_v_1.4 sodium channel complex (Figure 3D; Yan et al., 2017), resolved in the cryo- electron microscopy of channel protein nanoparticles. L5 turrets of Domains I, II and III (i.e., L5_I_, L5_II,_ and L5_III_) as well as L5 and L6 turrets of Domains III and IV, respectively, form highly negatively charged vestibules above the pore selectivity filter that serves as an electro-attractant for cation passage before reaching the selectivity filter below (Figures 3D,E). The eight conserved cysteines in extracellular turrets appear to be necessary for proper folding of 4 × 6 TM channels, as mutations of these conserved cysteines prevents their ion channel expression (Karmazinova et al., 2010). Varying from this conserved set of 8 extracellular turret cysteines in 4 × 6 TM channels are additional 1 to 3 cysteines in L5_II_ of Na_v_1 channels in vertebrates only (Stephens et al., 2015). Ca_v_3 T-type channels, but not Ca_v_1 or Ca_v_2 channels, possess extra cysteines – more than double the number beyond the core 8 cysteines in L5_I_ (+2), L5_II_ (from 0 to +5), L5_IV_ (+2), L6_IV_ (0 to +2), and S1-S2 in Domain I (+3) (Senatore et al., 2014). The positioning of numbers of cysteines and size of L5_II_ and L6_IV_ turrets is exploited to generate a variable ion selectivity from calcium to highly sodium selectivity, available in most invertebrates Ca_v_3 (T-type) channels by alternative splicing (Senatore et al., 2014).

## A Ubiquity in the Sugar Coating of the Extracellular Surface of Voltage-Gated Cation Channels at Glycosylated Amino Acid Residues

The external surface of voltage-gated channels are coated with (glycans) sugar groups, by an enzymatic, co- and post-translational process fundamentally critical for the proper folding and functional expression of voltage-gated ion channels (Baycin-Hizal et al., 2014). N-linked glycosylation involves sugars attached to the nitrogen atom of an asparagine amino-acid side chains (Mellquist et al., 1998) and is the most common form of glycosylation (Apweiler et al., 1999). O-linked glycosylation has a more promiscuous consensus binding site to different possible oxygen atoms of nascent proteins (Van den Steen et al., 1998), but has been reported for Na_v_1 channels (Ednie et al., 2015).

Glycosylation sites are enriched in the vicinity of the conserved cysteines on the longest L5_I_ and second longest L5_III_ extracellular loops of 4 × 6 TM voltage-gated cation channels. Electron microscopy structures of frozen sodium channel nano-particles resolves 20 and 16 sugars, incorporated into seven glycosylation sites on insect Na_v_1 (Shen et al., 2017) and electric eel Na_v_1.4 (Yan et al., 2017) sodium channels, with four and three glycosylation sites contained in L5_I_ and L5_III_ extracellular loops, respectively. While there are a conserved positioning of cysteines in extracellular loops, putative locations of glycosylation sites are not fundamentally shared between sodium and calcium channel families. Individual sodium (Na_v_2/Na_v_1), calcium channel (Ca_v_1/Ca_v_2) or T-type (Ca_v_3) channel genes can highly vary in their density of glycosylation sites. An often cited example of enrichment is in mammalian Na_v_1.4 sodium channel, which possesses four extra glycosylation site harbored in a unique ∼36 amino acid fragment added to the L5_I_ extracellular loop compared to non-mammalian, but vertebrate (e.g., eel) Na_v_1.4 (Bennett, 2002). Each sugar moiety contains a terminal sialic acid in N-glycosylation sites, contributing to the total electro-negativity of channel surface charge, with consequences to channel gating in biasing the voltage sensors by surface charge screening (Ednie and Bennett, 2012). The high electro-negativity due to glycosylation appears to reaches a saturation in mammalian Na_v_1.4, so that additional sialic acids contributed by the two known glycosylation sites of co-expressed mammalian beta subunit (Na_v_β_1,_ 25 kDa), influence channel gating on the more weakly glycosylated mammalian sodium channels (Na_v_1.2, Na_v_1.5, and Na_v_1.7) but not on channel gating of the heavily, saturated Na_v_1.4 channel with glycosylation sites (Johnson et al., 2004). The longest extracellular loops (L5_I_ and L5_III_) of the pore-forming α_1_ subunit (∼170 kDa) of Ca_v_1 and Ca_v_2 channels are preoccupied in cradling a large, mostly extracellular α_2_ subunit (∼150 kDa) (Leung et al., 1987). Almost all of the glycosylation sites (15 out of 16) in the resolved structure of the mammalian Cav1.1 channel complex are associated with the α_2_ subunit, with a single glycosylation site contained in L5_I_ extracellular loop of the pore forming α_1_ subunit of Ca_v_1.1 (Wu et al., 2016). Ca_v_3 T-type channels resemble Na_v_ sodium channels in lacking association with a large accessory subunit, and appear to possess more secondary structure with greater potential variations for glycosylation in their longer (L5_I_ and L5_III_) as well as L6_IV_ extracellular loops like the Na_v_ channels (Lazniewska and Weiss, 2017).

## The High Field Strength (Hfs) Site of the Pore Selectivity Filter Define the Sodium or Calcium Ion Selectivity in Eukaryotic Voltage-Gated Channels

**FIGURE 8:**
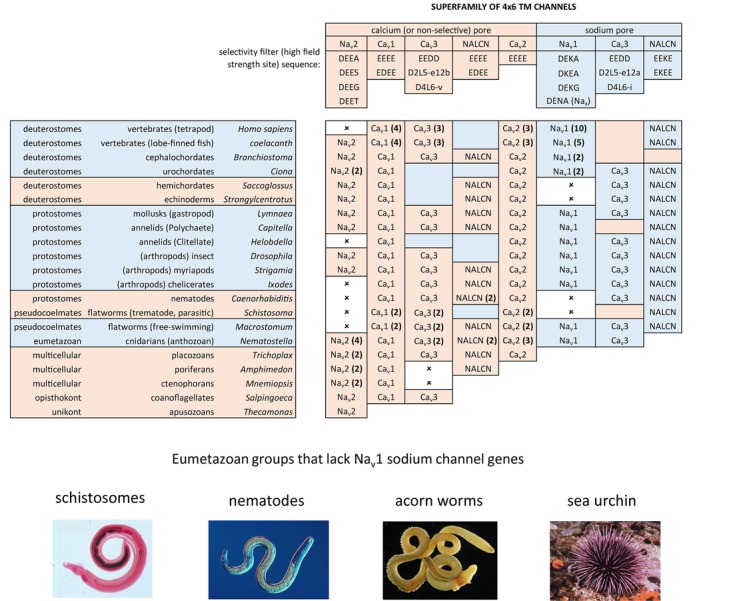
Illustration of the distribution of HFS site sequences in NALCN, sodium channels (Na_v_2 and Na_v_1) and calcium channels (Ca_v_1, Ca_v_2, and Ca_v_3) in different animals groups, as well as single cell eukaryotes *Thecamonas* (apusozoan) and *Salpingoeca* (choanoflagellate) (Top panel). Illustrations of animals (images imported from Wikipedia) lacking Na_v_1 channel genes (Bottom panel). Lifeforms with calcium-selective pores (shaded orange color) include HFS site sequences mostly of negatively charged glutamate (E) or aspartate (D) residues: DEEA, DEES, DEEG, DEET, EEEE, EDEE, NEEE, while sodium-selective pores (shaded blue color) have a lysine (K) residue in Domains II (EKEE and DKEA) or a lysine residue in Domain III (EEKE, DEKA, and DEKG). Ca_v_3 T-type calcium channels have a selectivity filter of EEDD and may be calcium or non-selective channels (shaded orange color), with high sodium selectivity engendered in invertebrate Ca_v_3 channels by alternative splicing of exon 12a coding for a novel L5_II_ turret (shaded blue color). Greater sodium selectivity also involves a D4L6i extracellular turret found in invertebrates instead of the D4L6v turret found in cnidarians and vertebrates. Numbers in brackets next to the gene indicate the number of gene isoforms (2, 3, 4, 5, or 10) that have been identified in a species, outside of one. An () indicates loss of gene in the indicated species. Amino acid sequences were aligned using MUSCLE 3.7 (Edgar, 2004) within EMBL-EBI web interface (Chojnacki et al., 2017).

A critical feature that emerges from the structural asymmetry in eukaryotic 4 × 6 TM channels is a highly changeable ion selectivity that ranges from highly calcium-selective channels (like Ca_v_1/Ca_v_2 channels) and highly sodium-selective channels (like Na_v_1 channels). The 4 × 6 TM calcium and sodium channels are at least a thousand-fold and ten-fold more selective for their particular cation, respectively, amongst competing native cations (e.g., Ca^2+^, Na^+^, and K^+^) (Hille, 1972; Hille, 2001; Finol-Urdaneta et al., 2014). The high calcium or sodium selectivity is largely converted by a singular, determinative residue at the most constricted point of the funnel-like pore known as the HFS site of the pore selectivity filter (Heinemann et al., 1992; Schlief et al., 1996). The HFS site is a negatively charged carboxylate, derived from a glutamate residue in the bacterial sodium channel pore of Na_v_Ab of TL**E**SWSM (Yue et al., 2002). The equivalent HFS site in eukaryotic calcium-selective channels is contributed by different amino acids from Domains I, II, III and IV, forming a ring of electronegative, glutamate residues configured as “EEEE” in Ca_v_1 and Ca_v_2 channels (Figure 8). The HFS site of sodium-selective channels are always differentiated with a positively charged lysine (K) residue configured in the third domain as “DE**K**A” in most Na_v_1 channels (Noda et al., 1984).

Native bacterial channels are not known to possess calcium-selective channels, but such a channel can be artificially created by engineering symmetrical rings of negatively charged carboxylates from aspartates (TL**DD**WS**D** from TL**ES**WS**M**) at twelve amino acid positions in the pore, spanning the HFS site glutamate residue, and downstream amino acids dubbed “Ca”, (position +1) and “Na”, (position +4). (Na_v_Ab mutant, Figure 4; Tang et al., 2014). The “Ca” and “Na” positions contribute to the outermost vestibule of the re-entrant pore selectivity filter, where they appear to be engineered to serve as potential “beacons” for attracting “Ca” or “Na” ions to the eukaryotic pore.

## The Uniquely Asymmetrical Pore Selectivity Filter Define the Sodium Ion Selectivity in Eukaryotic Voltage-Gated Channels

Molecular dynamics modeling illustrate how sodium selectivity is conserved in eukaryotic 4 × 6 TM channels involving four key amino acid residues (Zhang et al., 2018). The “*Na beacon*” position in Domain II (HFS+4) is always a glutamate residue (E) in known Na_v_2 and Na_v_1 channels, where it is optimally positioned in the outer vestibule to attract incoming cations (Zhang et al., 2018). The “*Na beacon*” relays incoming cations to the aspartate residue (DEKA) of the HFS in Domain I and then to the glutamate residue (DEKA) of the HFS site of Domain II, in a set of likely side-chain swinging transitions between these three negatively charged carboxylate residues in the pore selectivity filter (Zhang et al., 2018). The omnipresent, positively charged lysine residue of Na_v_1 channels of the HFS site (DE**K**A) protrudes into the spacious central cavity, repulsing the positive cations to the opposite side wall of the pore, where the ions are funneled, sequentially to the negatively charged (D and E) side chains of the HFS site (**D**EKA and D**E**KA) of the pore selectivity filter (Zhang et al., 2018). A critical structural deviation only found in Na_v_2 and Na_v_1 channels is a shortening of the selectivity filter in the second domain (Tikhonov and Zhorov, 2012), bringing the “*Na beacon*” residue in closer proximity to the other carboxylate site chains of the HFS site (D and E) residues required for coordination and selective passage of Na^+^ ions through the Na_v_1 channel pore (Shen et al., 2017). Homologous positioning of the pore selectivity filters and the Na site of 4 × 6 TM sodium channels are not easy to translate into sequence alignments with calcium channels or bacterial sodium channels. A single gap in amino acid sequence is inserted into multiple sequence alignments to convey this discrepancy for the 4 × 6 TM sodium channel pores (see Figure 4; Tikhonov and Zhorov, 2018).

Known variations in the HFS site of the pore selectivity filter of Na_v_1 channels including substitution of the 4th position from alanine (DEK**A**) to glycine (DEK**G**) identified in flatworm Na_v_1 channels (Jeziorski et al., 1997) and many alternative isoforms of duplicated Na_v_1 genes in teleost fish (Jost et al., 2008). The 4th position of the HFS site is not involved in coordinating ion selectivity, as predicted by modeling (Shen et al., 2017) and experimental evidence (Wu et al., 2013). The HFS lysine residue is relocated from Domain III (DE**K**A) in most metazoans to Domain II (D**K**EA) in cnidarians. The HFS site variation likely reflects a mirror imaging of selectivity filters swapped between Domain III and Domain II in cnidarian Na_v_1 channels without major consequences to their sodium selectivity (Spafford et al., 1998; Figure 8). Indeed, native sodium currents in cnidarians highly resemble mammalian Na_v_1 currents with a similar profile of ion selectivity for monovalent and divalent ions, suggesting that the cnidarian pore containing a DKEA HFS likely confers a similarly high sodium selectivity as the DE**K**A configuration of vertebrate Na_v_1 channels (Grigoriev et al., 1996; Spafford et al., 1996). The molecular constituents necessary for these channels to generate a high sodium selectivity hasn’t been assessed in cnidarian Na_v_1 channels, because these channels do not generate expressible ion currents *in vitro*, like other non-pancrustacean, invertebrate Na_v_1 channels (Schlief et al., 1996; Gur Barzilai et al., 2012).

The replacement of a positively charged lysine residue in the HFS site of Domain II or III with a negatively charged glutamate residue as D**EE**A differentiate the Na_v_2 channels, which are always lacking in sodium ion selectivity (Zhou et al., 2004). Na_v_2 channels otherwise closely resemble the Na_v_1 channels in structure and are distributed in genomes from single cell eukaryotes and multicellular animals including vertebrates, but not including ray-finned fish or tetrapods, like mammals (Zakon et al., 2017; Figure 8). Molecular modeling suggest a much wider and accommodating “DEEA” pore for Na^+^, K^+^, and Ca^2+^ ions in the absence of a protruding positively charged lysine residue, funneling ions to the side wall containing the carboxylate (D and E) side chains of the HFS site in Na_v_2 channels (Shen et al., 2017). Substitution of small amino acids (glycine, DEEG; serine, DEES; threonine, DEET) for the alanine in the 4th position of the “DEEA” HFS site occurs in Na_v_2 channels (Moran et al., 2015) in a homologous position as Na_v_1 channel substitutions in the 4th position of the HFS site. The omni-presence of the “*Na beacon*” in Domain II and the first and second position of “DE” within the HFS, reflects a common mode of ion selectivity for Na_v_2 and Na_v_1 channels, outside the uniquely protruding, positively charged lysine residue in the HFS site, which is required for limiting ion selectivity to sodium ions for Na_v_1 channels.

## A More Symmetrical Pore Selectivity Filter is Present in Calcium-Selective, Voltage-Gated Channels

A uniquely, more symmetrical, pore selectivity filter is shared amongst calcium channels, compared to 4 × 6 TM sodium channels. An aspartate (D) residue is ubiquitously located in the “Ca” position (HFS+1) of calcium channels (Ca_v_1, Ca_v_2, and Ca_v_3) (Figure 8) and dubbed a “*Ca beacon*” for its position just above the HFS site, in an opportune position to attract incoming calcium ions to the pore selectivity filter below (Tikhonov and Zhorov, 2011). The “*Ca beacon*” is located in the vicinity of the outer pore of Domain II as the “*Na beacon*” of sodium channels (Figure 4; Tikhonov and Zhorov, 2011). 4 × 6 TM calcium channels vary from 4 × 6 TM sodium channels in possess a pseudo-symmetry of their pore selectivity filters, more resembling the four-fold symmetry of bacterial 1 × 6 TM sodium channels. Calcium channels lack the shortened pore selectivity filter for Domain II found in sodium channels, and bear a more symmetrical, electro-negative ring of glutamate residues in the HFS site, configured as “EEEE” in Ca_v_1 and Ca_v_2 channels (Figure 8). The only known deviation of the “EEEE” configuration of the HFS site is the basal Ca_v_1 homolog isolated from *S. rosetta*, which retains a high calcium selective, even though the carboxylate side chain in Domain II is shortened by one carbon chain as “EDEE” (Mehta, 2016).

## Orphan Gene Nalcn Possesses Pore Selectivity Filters That Resemble Calcium and Sodium Channels

NALCN channels are usually a single representative in most animal groups, and possess variable HFS sites that resemble calcium channels “EEEE” (or rarely “EDEE”) or can resemble Na_v_1 sodium channels with a positively charged lysine (K) residue in Domain II or Domain III as EKEE or EEKE, respectively (Figure 8; Senatore et al., 2013). Many invertebrates groups possess a flexibility in generating alternative pores which resemble calcium- or sodium-selective NALCN channels specialized for different tissues (Senatore et al., 2013). A duplication of Exon 15 coding for region spanning the selectivity filter residue in Domain II of NALCN creates alternative calcium-selective (EEEE) and sodium-selective (EKEE) pores in the Platyhelminthes (flatworms) and protostomes of the lophotrochozoan lineage (mollusks and annelids), and non-chordate deuterostomes (echinoderms and hemichordates) (Figure 8; Senatore et al., 2013). A separate duplication event in Exon 31 of the Ecdysozoan lineage creates different alternative calcium-selective (EEEE) and sodium-selective (EEKE) NALCN pores that are retained in myriapods (includes centipedes, millipedes) and chelicerates (includes Arachnids like mites and ticks) (Figure 8; Senatore et al., 2013). NALCN channels are well described as a cation leak conductance that play roles in generating rhythmic behaviors in invertebrates and vertebrates (Cochet-Bissuel et al., 2014), while also highly curious channels in retaining both calcium selective and sodium selective pore structures in many non-vertebrates (Senatore et al., 2013).

## Ca_v_3 T-Type Channels have a Calcium-Like Pore Selectivity Filter That Can Generate High Calcium or Sodium Selectivity by Alternative Extracellular Loops Coding for L5_Ii_ and L6_Iv_

Ca_v_3 T-type channels have a HFS site in the selectivity filter that resembles calcium channels (EEEE), but notably different, and universally EE**DD**, shortened in carbon chains of the carboxylate side chain residues (glutamate (E) to aspartate (D) in Domains III and IV) compared to Ca_v_1 and Ca_v_2 channels (Perez-Reyes et al., 1998). The most calcium-selective, Ca_v_3 T-type channel, Ca_v_3.1, is more sodium permeable (∼1 Na^+^ per 5 Ca^2+^) (Shcheglovitov et al., 2007; Senatore et al., 2014) than Ca_v_1 and Ca_v_2 calcium channels (∼1 Na^+^ per 1,000 Ca^2+^), suggesting that the HFS site of EE**DD** and other contributing pore selectivity filter residues, support a less calcium selective pore in Ca_v_3 T-type channels. Invertebrates generate a high sodium selectivity in their Ca_v_3 T-type channels resembling Na_v_1 channels through alternative splicing of a novel cysteine-enriched L5_II_ extracellular turret coded in exon 12 (Senatore et al., 2014).

## Summary of Configurations of HFS Sites That Generate Sodium or Calcium Selective Pores

Na_v_, Ca_v_ and NALCN channels possess a universal set of HFS site configurations with calcium- or non-selective pores with electronegative residues in Domains II and III (E**EE**E, E**DE**E, E**ED**D, D**EE**A, D**EE**S, D**EE**G, and D**EE**T) and sodium-selective pores with a lysine in Domains II (D**KE**A, D**KE**G, and E**KE**E) or Domain III (D**EK**A and E**EK**E). More variable HFS pores are found in the 4 × 6 TM representatives outside of the animal/fungi supergroup of Unikonts (lifeforms with one flagella), such as diatoms, brown algae, oomycetes, cilates and dinoflagellates, and within branches of the Bikonts (lifeforms with two flagella), such as the green algae (chlorophytes and prasinophytes) (Verret et al., 2010). Approximately half of these non-animal representatives possess a HFS site resembling a calcium-selective pore (N**EE**E, E**EE**E, D**DD**D, and E**ED**E) (e.g., see representatives in Figure 7) and the rest of the pores are characterized by a pattern that represents experimentation and divergence. These pores may resemble a sodium-selective channel D**DK**D or possess HFS sites that are unlike any animal representative (e.g., RSDD, RADD, SESE, TDEE, and TEND) (Verret et al., 2010).

## Importance of Specific Asymmetrically Arranged Residues in the Selectivity Filter for Pore Binding Ttx Binding

The outermost position “Na” (HFS site +4) is populated to form a negative-charged ring in the uppermost pore in many of the pore domains of eukaryotic sodium and calcium channels (Shen et al., 2018; Figure 4). The negatively charged aspartate (D) of the “Na” site in Domain IV is a frequently altered residue in animals adapted for resistance to pore-blocking, Na_v_1 channel toxin tetrodotoxin (TTX) (see TTX insensitive Na_v_1.4, Figure 4; Feldman et al., 2012). TTX is a bacterial toxin produced commonly by *Vibrio alginolyticus* which exists symbiotically within many discovered hosts, including notably pufferfish and many other fish species, tubellarian flatworms, blue-ringed octopus, western newt, toads, sea stars, angelfish, polyclad flatworms, Chaetognath arrow worms, nemertean ribbon worms and xanthid horseshoe crabs (Chau et al., 2011). A striking example of adaptation in the sodium channel pore is illustrated in the TTX-resistance of garter snakes (*Thamnophis sirtalis*) in districts where toxic prey newts (*Taricha granulosa*) are present, but not in the same species of garter snake in neighboring districts where these newts are absent (Geffeney et al., 2005). Similar residue changes in the “Na” position also provides for resistance to the analog saxitoxin (STX), contributing to the paralytic shellfish toxin, produced in toxic algal blooms in marine environments (Terlau et al., 1991). The many marine invertebrates that are completely resistant to TTX or STX, can comes at a cost of a lowered sodium ion selectivity in their Na_v_1 sodium channels (Grigoriev et al., 1996). TTX is a primary tool for the discrimination of Na_v_1 channels currents in doses ranging from the nanomolar to micromolar concentrations (Toledo et al., 2016). Nonetheless, the outer pore-blocking mechanism of TTX or STX or the micro-conotoxins (μ-CTXs) from venomous cone snails, is more the exception than the rule to how animal toxins regulate Na_v_1 channels. The vast majority of animal toxins are gating modifiers which take advantage of the greater structural variance outside of the outer pore of different Na_v_1 channels (Kalia et al., 2015). Another form of blockade are the frequency-, use- or state-dependent drugs that inhabit the aqueous vestibule within the channel pore, at higher affinities in particular channel states, such as when the inactivation gate is closed (Hille, 2001). Na_v_1 channels possess a much smaller aqueous vestibule within the pore for harboring drugs (e.g., local anesthetics like lidocaine) (Shen et al., 2017) compared to Ca_v_1 channels which possess a more accommodating pocket within the pore for harboring drugs (e.g., nifedipine, verapamil, diltiazem) (Wu et al., 2016). Both the bacterial Na_v_ channels and eukaryotic Na_v_ and Ca_v_ channels possess side-portals or “fenestrations” between domains, providing an access pathway of small hydrophobic drugs to the inside of the pore without required passage through the pore selectivity filter during channel pore opening (Catterall and Swanson, 2015).

## Origin of Na_v_2 Channels in Single Cell Eukaryotes

**FIGURE 9:**
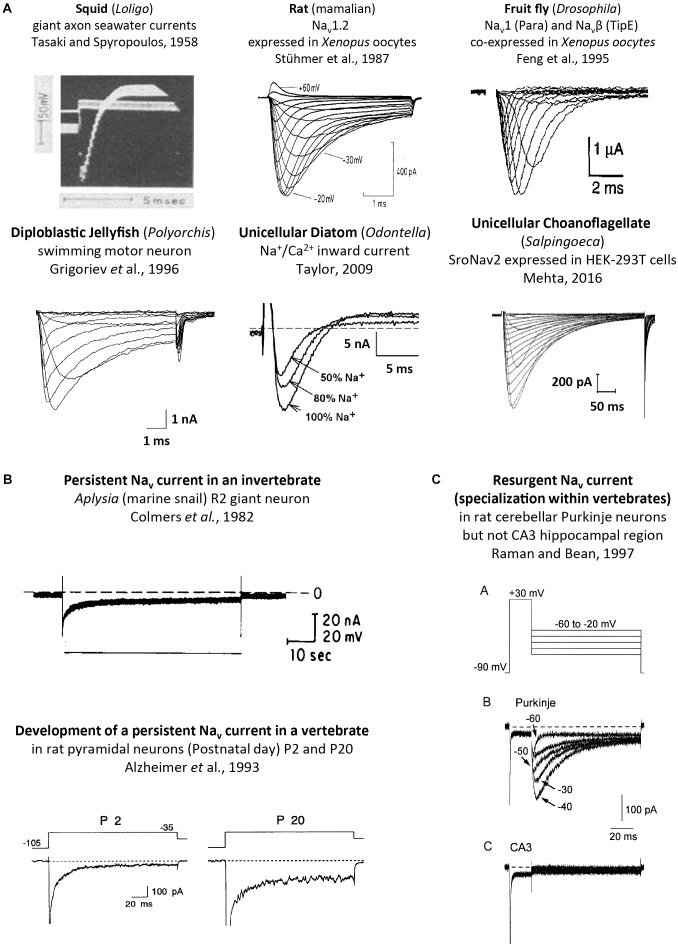
Sodium ion containing currents recorded in voltage-clamp studies of cells or expressed genes derived from single cell eukaryotes, invertebrates and vertebrates. **(A)** Squid, rat, fruit fly, jellyfish, diatom and choanoflagellates, reproduced with permission from Tasaki and Spyropoulos (1958), Stühmer et al. (1987), Feng et al. (1995), Grigoriev et al. (1996), Taylor (2009), Mehta (2016). **(B)** Persistent sodium currents in both invertebrates (top panel) and vertebrate neurons (bottom panel), reproduced with permission from Colmers et al. (1982) and Alzheimer et al. (1993), respectively. **(C)** Resurgent sodium currents, a property associated only with particular vertebrate neurons, reproduced with permission from Raman and Bean (1997).

Single cell eukaryotes, like diatoms generate an animal-like, rapid action potential spike of a few ms in length from a resting membrane potential of ∼-68 mV (Taylor, 2009). The action potential in diatoms is generated with a fast inward current carried by sodium and calcium ions, peaking at -∼ 20 mV, and recovering quickly from inactivation (τ = ∼7 ms) (Taylor, 2009). While diatoms functionally generate an animal-like sodium and calcium current (Figure 9A), structurally it can only be derived from a 1x6TM bacterial sodium channel gene or a 4 × 6 TM template with an architecture distantly resembling calcium channels from animals (Verret et al., 2010; Figure 7). The diatoms belong to the Heterokonts (multiple different shaped flagella), which are separate from the Unikonts (single or no flagella), which are the lineage which includes the animals. The most basal extant representative of a recognizable homolog to animal genes is a 4 × 6 TM Na_v_2 homolog found in an apusozoan, represented by *Thecamonas trahens* (Figures 1, 8; Cai, 2012). Apusozoans are Unikonts that lies outside the Opisthokonts (bearing a posterior flagella) that includes the choanoflagellates, animals and fungi (Cavalier-Smith and Chao, 2010). Choanoflagellates (posterior flagella; possessing ciliated collars like sponge choanocytes) are closer relatives to the animals and fungi than the apusozoans. Choanoflagellates are represented by *S. rosetta* which possess a Na_v_2 homolog, *Sro*Na_v_2 (Mehta, 2016), but also have expressible homologs for Ca_v_1 and Ca_v_3 channels too (Figure 1). Expression of *Sro*Na_v_2 channel from this extant representative of an earliest branching single-celled organism, reveals a non-selective ion channel, equally passing Na^+^, K^+^, or Ca^2+^ ions, and also possesses extremely slow gating kinetics (Figure 9A; Mehta, 2016). Gating of a typical sodium channel from the classical squid giant axon is akin to mammalian neurons is rapid, enabling action potential spikes of 1–2 ms in length (Figure 9A; Hodgkin and Huxley, 1952). *Sro*Na_v_2 is more than tenfold slower, taking more than 10 ms to fully activate, with a slow inactivation decay (τ of >50 ms), requiring ∼ more than 1/2 s to completely inactivate, and ∼12 s to fully recover from inactivation in a whole cell patch clamp recording of currents recorded in HEK293T cell lines (Mehta, 2016).

## Na_v_2 Channels have Functions Associated With Olfactory Sensing in Invertebrates

Na_v_2 homologs have also been recorded from arthropods such as cockroach (Zhou et al., 2004), honeybee (Gosselin-Badaroudine et al., 2016) and fruit fly (Zhang et al., 2011) as well as sea anemone (a cnidarian) (Gur Barzilai et al., 2012), the latter of which has four Na_v_2 channel homologs. Single Na_v_2 channel homologs have been identified in vertebrates species, including cartilaginous fish (Australian ghostshark), lobed-finned fish (coelacanth), and jawless fish (lampreys) (Zakon et al., 2017). All expressed Na_v_2 homologs possess slow gating kinetics, and are non-selective channels passing calcium and sodium ions (Gosselin-Badaroudine et al., 2016). The Na_v_2 channel from honeybee is unique in generating mostly a calcium current, contributed by a smaller-sized sodium current (Gosselin-Badaroudine et al., 2016). The honeybee Na_v_2 channel may be mostly a calcium current but does not possess the nearly exclusive (1,000:1) calcium over sodium ion selectivity characteristic of Ca_v_1 or Ca_v_2 calcium channels (Hille, 2001).

Na_v_2 channels are represented from single cell choanoflagellates to vertebrates mostly as a single gene, but it is not a required gene in every species. Na_v_2 channel genes appear to be completely lacking in yeast, flatworms, nematodes, chelicerates (within the arthropods) and clitellates (within the annelids), and also lacking in many vertebrates, including ray-finned fishes and tetropods, including mammals (Figure 8). The absence of either sodium (Na_v_2 and Na_v_1) channel genes in different animal species is in contrast with calcium channels (Ca_v_1, Ca_v_2, and Ca_v_3) which are omni-present, and not known to be secondarily lost in any animal group.

A possible function associated with Na_v_2 channels has been inferred from gene knockdowns in fruit fly (*Drosophila*), which possess a diminished ability to smell (Kulkarni et al., 2002), and a hyperkinetic mobility that is intensified by heat shock and starvation (Zhang et al., 2013). Na_v_2 channels have increased in gene number beyond a single homolog in urochordates such as *Ciona intestinalis* (two genes) ctenophores, sponges and placozoans (two genes), and as many as four genes in anthozoan or scyphozoan cnidarians (Figure 8). The diversification of Na_v_2 channels particularly within animals lacking true nervous systems (ctenophores, sponges and placozoans) or possessing vestigial nervous systems (tunicates), is suggestive of an expansion of roles for Na_v_2 filling of niches in the absence of animals with a mature nervous system. All vertebrate Na_v_2 channels identified in non-teleost fish, for example are only expressed outside of the nervous system (Zakon et al., 2017).

## Requirements for Nervous Systems was the Impetus for the Appearance and Retention of Na_v_1 Channels in Eu-Metazoans

Unicellular organisms (yeast, protozoans) and the earliest diverging multicellular animals, such as ctenophores (comb jellies), sponges and placozoans (*Trichoplax adhaerens*) lack Na_v_1 channels (Figure 8). These early branching lifeforms such as sponges (Leys, 2015) or placozoans (Senatore et al., 2017) have a “toolkit” of many of the building blocks for nervous systems, without possessing eu-metazoan nervous tissue. Ctenophores have a nervous system, but are lacking most of the classical transmitter receptors and ion channels, and other neuronal elements common to the eu-metazoan nervous system template (Moroz and Kohn, 2016). Inward currents generating membrane excitability containing calcium ions as a charge carrier is limited because of competing roles of calcium ions as an exquisitely sensitive intracellular messenger. Calcium ions are toxic to cells when levels rise too high and calcium ions have a propensity to precipitate with phosphate (as bone matrix) (Hille, 2001). Free concentrations of intracellular calcium is necessarily kept at very low (nM) within cells by intracellular calcium buffering, compartmental storage of calcium in intracellular organelles, and extrusion to the cell exterior (Hille, 2001). Sodium ions are relatively inert, maintained at high (mM) concentrations within cells, and serves as a major ion in generating osmotic pressure (Hille, 2001) and as a workhorse in the secondary active transport of hydrogen ions or amino acids (Hille, 2001). A key step in the appearance of nervous systems, was the amino acid changes in the HFS site and surrounding residues, allowing for the sodium-selectivity of Na_v_1 channels, and an ability to exploit the steep, calcium ion free electro-chemical gradient, for generating rapid, mostly inert, action potential spikes along nervous tissue.

## Singleton Na_v_1 Channels in Invertebrate Species are Functionally Limited to Nervous Systems

The cnidarians are the simplest eu-metazoans with Na_v_1 channel genes and they also are the simplest organism to possess a true nervous systems (Spafford et al., 1998). Cnidarians have a simple body plan of two germ layers (diploblastic), are lacking a coelom, and are radially symmetrical animals without cephalization (a brain localized in the anterior position) (Zapata et al., 2015). The Na_v_1 sodium channels in these basal species possess motor neurons which regulate the pulsating contractions of the jellyfish bell during swimming, are almost indistinguishable from those of the giant axons of the Atlantic squid or frog sciatic nerve, in their rapid millisecond gating and high sodium selectivity (Grigoriev et al., 1996). Sodium-selective Na_v_1 channels remain as a singleton gene in most invertebrates with functional currents limited in detection to nervous tissue, and lacking in functional protein expression in heart muscle or striated (skeletal-like) muscle or glands. mRNA coding for invertebrate Na_v_1 channels can be detected outside the nervous system, e.g., (Hong and Ganetzky, 1994; Gosselin-Badaroudine et al., 2015), but sodium currents have never been resolved outside the nervous system in known protostome invertebrates from the diverse lophotrochozoans to the ecdysozoans, e.g., (Hagiwara et al., 1964; Mounier and Vassort, 1975; Salkoff and Wyman, 1983; Brezina et al., 1994; Collet and Belzunces, 2007; Senatore et al., 2014). The primary expression of Na_v_1 sodium channel, “*para*” is in insect nervous systems, where *Drosophila* flies with *Para* locus mutations exhibit a temperature-sensitive paralyzes from nerve conduction blockade (Loughney et al., 1989). A niche left from a lack of Na_v_1 sodium channels outside of the nervous system can be filled with a sodium-selective Ca_v_3 T-type channel isoform, generated by the unique alternative splicing in the extracellular turret upstream of the Domain II pore in most invertebrates (Senatore et al., 2014). Presence of an alternative 12a exon engenders Ca_v_3 T-type channels with sodium selectivity (Figure 8), and is the only splice isoform found in the molluscan heart. The exon 12a containing T-type sodium channel isoform is almost ubiquitously present isoform in all eu-metazoans with organ-containing body cavities (coelomates), including some flatworms (pseudocoelomates) (Senatore et al., 2014).

Vertebrates underwent an expansion of Na_v_1 channel gene numbers to ten alongside the dramatic changes in body plan that include a greater sophistication of tissues, with different Na_v_1.x isoforms expressed at high density in particular tissues such as brain (e.g., Na_v_1.6), heart (e.g., Na_v_1.5) and skeletal muscle (Na_v_1.4) (Zakon et al., 2017).

## Na_v_1 Channels are Lost in Many Invertebrate Groups, Compared to an Apparent Ubiquity in the Retention of Nalcn and Calcium Channel Genes

A loss of Na_v_1 sodium channel genes is also common in non-vertebrate animals. For example, Na_v_1 sodium channels are present in free living flatworms (e.g., *Schmidtea mediterranea*) but are lacking in parasitic flatworms (*Schistosoma*). Na_v_1 channels are also lacking in nematodes, approximately half of animals in this phyla which are parasitic (Zhang, 2013; Figure 8). Nematodes lack both Na_v_2 and Na_v_1 channels, and possess simple, vestigial nervous systems that may related to their often obligate parasitism of host species (Zhang, 2013; Figure 8). All-or-none action potentials of body wall muscles requires inward Na^+^ influx in nematodes (Liu et al., 2011), but it is likely derived from other sources such as sodium-selective Ca_v_3 T-type channels, *in lieu* of Na_v_2 and Na_v_1 channels (Senatore et al., 2014). Deuterostomes such as the echinoderms (e.g., *Strongylocentrotus purpuratus*) and their relatives the hemichordates (e.g., *Saccoglossus kowalevskii*) have sparse, radially symmetrical nervous systems and also lack Na_v_1 channel genes (Figure 8). In comparison, genes coding for all three calcium channels (Ca_v_1, Ca_v_2, Ca_v_3) and NALCN is present in every known extant, eu-metazoan to date (Senatore et al., 2016), while the rise and fall of Na_v_1 channel genes (from zero to ten) appears to relate to a species’ necessity for sodium dependent action potentials, a vital feature of metazoan nervous systems (Figure 8).

## Na_v_2 Channels Differ from Na_v_1 Channels in Lacking Elements for Sodium Ion Selectivity and Fast Gating

**FIGURE 10:**
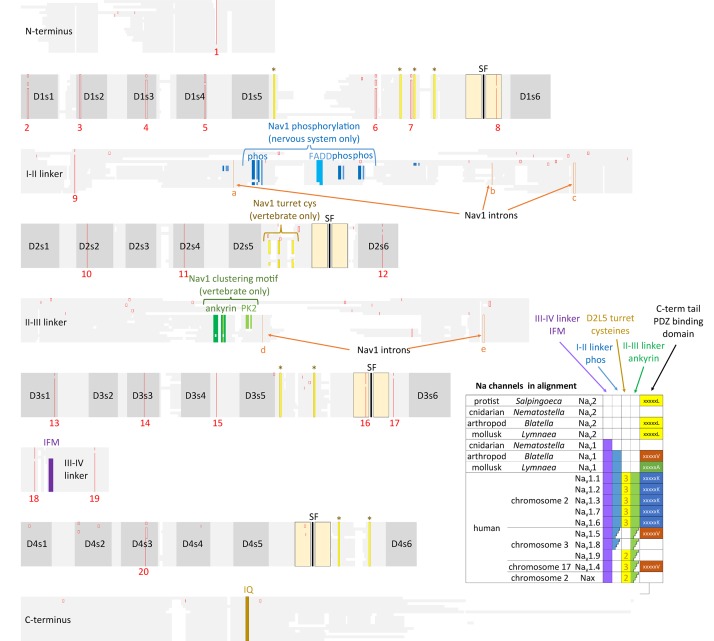
Amino acid alignments illustrating the conservation of twenty intron splice sites (red vertical lines) shared between Na_v_2 channels and Na_v_1 channels. The close kinship between the Na_v_2 channel class only found in non-vertebrates and the classical Na_v_1 channels is evident in the shared genomic structure. Na_v_1 differ primarily from Na_v_2 channels in containing three additional intron splice sites (a–c) in the I-II linker and two splice sites (d,e) in the II-III linker. Extra exons in the I-II linker provides exons coding for conserved phosphorylation sites within invertebrate Na_v_1 and vertebrate homologs (Na_v_1.1, 1.2, 1.3, 1.6, and 1.7) expressed in central nervous systems. Vertebrate, but not invertebrate Na_v_1 channel homologs contain three extra L5_II_ turret cysteines, and an ankyrin binding site that contribute to the localized clustering of Na_v_1 channels in vertebrate Axon Initial Segments and nodes of Ranvier (Hill et al., 2008). Na_v_1 channels possess an IFM motif and surrounding residues within the III-IV linker which contribute to the fast inactivation (West et al., 1992). Na_v_2 and Na_v_1 channels possess a conserved proximal C-terminus containing an IQ motif required for calmodulin binding (Ben-Johny et al., 2014, 2015). Different C-terminal motifs containing PDZ binding domains are possessed in sodium channels. Amino acid sequences were aligned using MUSCLE 3.7 (Edgar, 2004) within EMBL-EBI web interface (Chojnacki et al., 2017).

The heritage of the first Na_v_1 channels emerging from Na_v_2 channel common ancestors is evident in the shared genomic structures of Na_v_1 and Na_v_2 channel genes. 20 intron splice sites are homologously shared in Na_v_1 and Na_v_2 channels, and these are all the intron splice sites spanning the conserved pore and voltage sensor domains (Figure 10). The same conservation of intron splice sites is paralleled within the calcium channel family, where Ca_v_1 and Ca_v_2 channels possess 31 shared intron splice sites. Both the Na_v_1 and Ca_v_2 channels emerged from primordial Na_v_2 and Ca_v_1 channel ancestors under a selection pressure for the evolution of specialized nervous tissue. Emergent roles of Na_v_1 and Ca_v_2 channels appeared for fast conduction along nerve axons, and secretion of neurotransmitters between nerve synapses, respectively. The pairs of ion channel homologs in the most basal extant organisms known including Na_v_2/Na_v_1 in cnidarians (Gur Barzilai et al., 2012), or Ca_v_1/Ca_v_2 channels in placozoan, *Trichoplax adhaerens* (Senatore et al., 2016), are not easily distinguished from each other, as one would expect in extant organisms which are phylogenetically closer to the common ancestor containing the first Na_v_1 channels or Ca_v_2 channels.

A defining feature that ubiquitously separates Na_v_1 and Na_v_2 channels is the lysine residue in Domain II (cnidarians) and III (all others metazoans) of the HFS site in the selectivity filter that confers the high sodium selectivity of Na_v_1 channels (Heinemann et al., 1992; Schlief et al., 1996). With substitution of the glutamate residue for a lysine residue D**K**EA or DE**K**A HFS site in Na_v_1 for the glutamate (E) in the DEEA selectivity filter of Na_v_2 channel of single-cell choanoflagellate, *S. rosetta*, the Na_v_2 channel transforms from being non-selective for calcium and sodium ions to exclusively selective for sodium ions (Mehta, 2016), resembling the Na_v_1 channels found in animals with nervous systems.

## Na_v_1 Channels Expanded in Gene Numbers in Vertebrates to Fill Novel Tissue Niches

Sodium channels expanded from one gene (the condition in invertebrates) to ten vertebrate genes by gene duplication (Figure 1), and this heritage can be traced by their linkage to HOX (homeotic) developmental genes (Lopreato et al., 2001). Vertebrate sodium channels Na_v_1.1, Na_v_1.2, Na_v_1.3 and Na_v_1.7, are expressed primarily in central and peripheral nervous systems and are coded on human chromosome number 2 (linked to HOX D gene) (Lopreato et al., 2001). Na_v_1.6 is the primary sodium channel found in vertebrate myelinated axons at Nodes of Ranvier, is coded on chromosome number 12, and is linked to HOX C gene (Lopreato et al., 2001). Na_v_1.8 and Na_v_1.9 are expressed in dorsal root ganglia, and Na_v_1.5 is primarily expressed in heart muscles (Lopreato et al., 2001). Na_v_1.5, Na_v_1.8, and Na_v_1.9 are clustered on chromosome 3, linked to Hox A (Lopreato et al., 2001). Na_v_1.4 is specialized to vertebrate skeletal muscle, and is found on chromosome 17, linked to Hox B (Lopreato et al., 2001). A tenth vertebrate sodium channel, Nax, (located on chromosome 2) is the most diverged in sequence, is lacking voltage-sensitive gating and plays a likely role as a putative salt sensor in the subfornical organ (Noda and Hiyama, 2015).

## Specializations Within Cytoplasmic Regions (Linkers And Termini) of Four Domain Channels are Not Evident in Cngk Channels

A consequence of chaining of domains together in the four domain structure of the 4 × 6 TM channels is that it generates opportunities for divergence and specialization within the differing voltage-sensor and pore domains. It creates unique extensions of differing extracellular turrets or intracellular linkers linking the four domains, and a trimming down to singleton, asymmetrically arranged, amino- and carboxyl-termini in 4 × 6 TM channels compared to the four N- and C- terminal tails in the repeating four subunits composing the 1x6TM potassium channels (Long et al., 2005) or bacterial sodium channels (Payandeh et al., 2011) or (Figure 2). Cyclic nucleotide-gated potassium channel (CNGK) channels are a 4 × 6 TM channel representative amongst the potassium channel superfamily. CNGK channels lack the uniquely different extracellular turret extensions in differing domains or asymmetrical intracellular linkers between domains that are characteristic of 4 × 6 TM sodium or calcium or NALCN channels (Bonigk et al., 2009). CNGK channels possess a pseudo-symmetry of four nearly identically repeating domains, linked together by equally short and repeating, cytoplasmic linkers and extracellular regions. There is a similarly positioned cyclic nucleotide binding domain (CNBD) within each of their four domains [Bonigk et al., 2009; Fechner et al., 2015]. The CNGK channels appears to be ancient like the lineage of 4 × 6 TM calcium channels with representatives in single cell choanoflagellates (Fechner et al., 2015), in marine invertebrates (Bonigk et al., 2009) and in vertebrates (fish) (Fechner et al., 2015). There appears to be no obvious adaptations or specializations in the four domains of CNGK channel homologs, despite their apparent ancient history. CNGK channels contain four repeating subunits linked together, but only the sodium, calcium and NALCN channel lineage adopted a particular asymmetrical architecture.

## The III-IV Linker and Proximal C-Terminal IQ Domain Represent a Globular Domain Which Regulates Calcium- and Voltage-Sensitive Gating

The most constrained of the cytoplasmic linkers is the III-IV linker which is always shorter than the I-II and II-III linkers in 4 × 6 TM channels (Figure 2B), and limited in size to mostly 53 to 57 amino acids (Stephens et al., 2015). The exception is the unusual optional splicing of inserts (e.g. exon 25C) in invertebrate and vertebrate Ca_v_3 T-type calcium channels (Senatore and Spafford, 2012). Also highly conserved is a calcium-calmodulin binding IQ domain within the first 155 aa downstream of the proximal C-terminus in Ca_v_1, Ca_v_2, Na_v_1 and Na_v_2 channels, but is absent in Ca_v_3 T-Type channels or NALCN channels (Figures 7, 10). The calmodulin binding in the C-terminus puts a calcium ion sensor within the pathway of calcium entry through the channel pore of calcium channels (Lee et al., 1985; Zuhlke et al., 1999) and resides in a similar position in sodium channels (Mori et al., 2000; Potet et al., 2009; Sarhan et al., 2009) to potentially regulate channel gating. The conserved III-IV linker and the C-terminal domain join to form a globular domain in the cryo-electron microscopy of single calcium channel (Wu et al., 2015, 2016) and sodium channel (Shen et al., 2017; Yan et al., 2017) nanoparticles.

The globular domain formed by the III-IV linker and proximal C-terminus generates a qualitatively different regulation of gating in different calcium and sodium channels, based on sequence differences. A highly conserved IQ motif among L-type channels contributes to a canonical calcium-sensitive inactivation shared amongst invertebrate Ca_v_1 and vertebrate Ca_v_1.2 and Ca_v_1.3 channels (Taiakina et al., 2013). This conservation extends to single cell protozoans, including *Paramecium* L-type currents which possesses a calcium-calmodulin dependent inactivation (Brehm et al., 1980). A less conserved proximal C-terminal IQ motif engenders a calcium-dependent facilitation of vertebrate Ca_v_2.1 and Ca_v_2.2 channels involving calmodulin kinase activation (Christel and Lee, 2012). A calcium dependent facilitation and relief of G-protein βγ subunit inhibition by repetitive nerve activity are uniquely featured in vertebrate synaptic Ca_v_2 calcium channels (Zamponi and Currie, 2013), and such modulation is lacking in homologous invertebrate Ca_v_2 channels (Dunn et al., 2018). Invertebrates Ca_v_2 channels are regulated by G-protein coupled receptor signaling that involves a non-voltage dependent form of regulation of activity involving phosphorylation by SRC kinase (Dunn et al., 2018) or cAMP dependent protein kinase A (Huang et al., 2010).

All sodium channels have a highly conserved proximal C-terminal IQ motif, including Na_v_2 channels in single cell, choanoflagellate Na_v_2 (Figure 10; Mehta, 2016). The proximal C-terminal IQ motif contributes to a calcium-sensitive regulation that is variable amongst different vertebrate Na_v_1 channels (Mori et al., 2000; Potet et al., 2009; Sarhan et al., 2009), with a potential secondary binding site for calmodulin in the III-IV linker (Sarhan et al., 2009, 2012). Ca_v_3 T-Type channels differ from other calcium and sodium channels in possessing an equivalent high affinity binding site for calmodulin at their unique “gating brake” located in the proximal I-II linker at homologous location where the accessory Ca_v_β subunits normally associate with Ca_v_1 and Ca_v_2 channels (Chemin et al., 2017).

## Fast, Voltage-Sensitive Gating is Contributed by the Iii-Iv Linker in Na_v_1 Channels

Na_v_1 sodium channels are differentiated from calcium channels in possessing a very rapid gating that is required for generating high frequency trains of action potential spikes milliseconds in length, observed in nervous systems (Hodgkin and Huxley, 1952). Faster sodium channel gates contribute to a faster overshooting action potential, while slower and higher voltage-activated calcium channels contribute to a plateau potential following the overshoot, such as in the vertebrate ventricular action potential (Hille, 2001).

A key player which differentiates fast Na_v_1 channel gating is in the III-IV linker (Ulbricht, 2005) (Figure 10). Glycine residues flanking the III-IV linker create a flexible hinge, permitting movement of the III-IV linker, and rapid inactivation, within milliseconds of sodium channel opening (Kellenberger et al., 1997). A key “IFM” motif in the center of the III-IV linker, serves as a critical “latch” for the “hinged lid” (West et al., 1992), The IFM motif forms a plug inserting in the corner surrounded by the outer S4-S5 and inner S6 segments in repeats III and IV in three dimensional structure, indicating a likely allosteric blocking mechanism for fast inactivation gating in Na_v_1 channels (Yan et al., 2017). The “IFM” motif, and its surrounding conserved sequence of the III-IV linker is absent in Na_v_2 channels, and appears to be a critical structural feature responsible for the slower inactivation gating of Na_v_2 channels in choanoflagellates, according to our mutagenesis studies (Mehta, 2016). Other fine tuning for fast channel gating in Na_v_1 channels include likely changes to the speed of voltage-sensor gating charge movements and the coupling efficiency of the voltage-sensors to pore gates (Capes et al., 2013), as well as roles for the proximal C-terminus which includes a calcium sensor in a calmodulin-binding IQ motif (Christel and Lee, 2012), found in sodium and calcium channels.

## Na_v_1 Channels from Protostome Invertebrates and Those Expressed in Vertebrate Nervous Systems Share Protein Kinase Phosphorylation Sites in the I-II Linker

**FIGURE 11:**
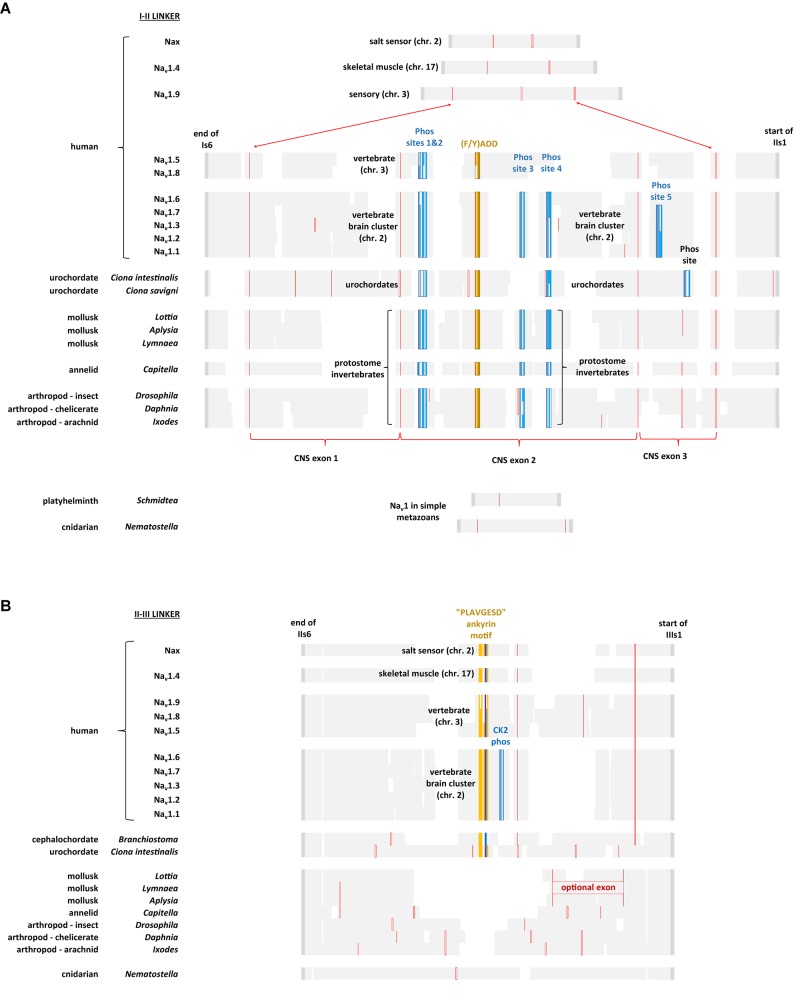
Alignment of amino acid sequences coding for cytoplasmic I-II linker **(A)** and II-III linker **(B)** of invertebrate and vertebrate Na_v_1 channels. Note the expanded size of the cytoplasmic I-II linker in protostome invertebrate Na_v_1 and vertebrate brain cluster channels (Na_v_1.x, where x = 1,2,3,6, and 7) containing four conserved phosphorylation sites at serine residues. Two of these four phosphorylation sites are subject to phosphorylation in mammalian Na_v_1.2 channels by cAMP dependent protein kinase A (serine: 573 and 623) (Smith and Goldin, 1996) and one of the four is a protein kinase C regulatory site (serine 576) (Cantrell et al., 2002). Other vertebrate sodium channels, such as Na_v_1.5 and Na_v_1.8 retain two out of four of these phosphorylation sites (serine: 573 and 576) while Na_v_1.4, Na_v_1.9 and Nax lack any of these phosphorylation sites. While all chordate Na_v_1 channels possess the ankyrin G binding motif, only the nervous system specific cluster of vertebrate sodium channels (Na_v_1.1, Na_v_1.2, Na_v_1.3, Na_v_1.6, and Na_v_1.7) possess a CK2 serine/threonine kinase phosphorylation site downstream of the ankyrin G binding site in the II-III linker, that greatly increases affinity and required for the formation of stable ankyrin G – sodium channel complexes upon phosphorylation (Xu and Cooper, 2015). Amino acid sequences were aligned using MUSCLE 3.7 (Edgar, 2004) within EMBL-EBI web interface (Chojnacki et al., 2017).

The region of greatest sequence divergence between Na_v_2 and Na_v_1 channels is in the cytoplasmic I-II and II-III linkers, and these differences appear to relate to the increasing specialization of Na_v_1 channels for more sophisticated signaling, that reaches its apex in vertebrate tissues (Figures 10, 11). Cytoplasmic regions linking the four domains created in the first 4 × 6 TM channels, diverged and specialized for the more complex and diverse tissue environments for different genes isoforms as numbers grew from singleton gene homologs of the simplest single cell organisms to the ten sodium and ten calcium channel genes available in the vertebrate body plan.

With increasing animal complexity, there is adjustments and fine-tuning of cellular function within signaling networks involving neurotransmitters, hormones and other modulators to up-and-down regulate cellular activity and excitability. One of the cellular targets for regulation is Na_v_1 channels, both in their control of gene expression and changes to ion channel gating. The Na_v_1 channel in protostome invertebrates, but not those in simple metazoans (e.g., cnidarians, flatworms) possess a greatly expanded cytoplasmic I-II linker (coded in exon 11), containing four conserved phosphorylation sites at serine residues (Figure 11A) (Fux et al., 2016). Two of these four phosphorylation sites are subject to phosphorylation in mammalian Na_v_1.2 channels by cAMP dependent protein kinase A (serine: 573 and 623) (Smith and Goldin, 1996, 1997) and one of the four is a protein kinase C regulatory site (serine 576) (Cantrell et al., 2002) (Figure 11A). Phosphorylation by protein kinase A (Li et al., 1992) or protein kinase C (Numann et al., 1991), downregulates sodium channel activity. These phosphorylation sites are shared amongst the single Na_v_1 channel homolog expressed in nervous systems of protostome invertebrates and in the cluster of related sodium channels specifically expressed in the vertebrate central and peripheral nervous systems, Na_v_1.1, Na_v_1.2, Na_v_1.3, Na_v_1.6, and Na_v_1.7 (Figure 11A; Fux et al., 2016). Other vertebrate sodium channels, such as Na_v_1.5 and Na_v_1.8 retain two out of four of these phosphorylation sites (serine: 573 and 576) while Na_v_1.4, Na_v_1.9, and Nax lack any of these phosphorylation sites (Figure 11A). The conservation of sequences for neurotransmitter and G protein coupled regulation of sodium channel activity supports the hypothesis that Na_v_1 channels evolved in conjunction with appearance of nervous tissue, with a closer structural kinship of the nervous system specific (Na_v_1.1, Na_v_1.2, Na_v_1.3, Na_v_1.6, and Na_v_1.7) sodium channels in vertebrates, with the singleton nervous system-specific Na_v_1 sodium channel within the protostome invertebrates.

## Ankyrin-G Binding in the II-III Linker as a Vertebrate Specialization for Clustering Sodium Channels

Further specialization of sodium channels for nervous systems is evident in the emergence of unique sequences in chordate Na_v_1 channels, related to the specific targeting and clustering of sodium channels to Axon Initial Segments (AIS) and Nodes of Ranvier (Hill et al., 2008). The singleton Na_v_1 channels in basal chordates including the urochordates (e.g., *C. intestinalis*) and cephalochordates (*Branchiostoma floridae*) and the ten vertebrate Na_v_1 channels, share a conserved anchoring motif that binds ankyrin G, an adapter protein that tethers the Na_v_1 channels and KCNQ2/3 potassium channels to the spectrin-actin cytoskeleton (Hill et al., 2008). The anchoring motif is a 9 amino acid sequence V/A-P-I/L-A-x-x-E-S/D-D, where x can be any amino acid (Hill et al., 2008). The ankyrin G binding motif is located in the II-III linker, a cytoplasmic linker that is ∼50% shorter than the I-II linker of vertebrate neuronal Na_v_1 channels (Figures 10, 11B). While all chordate Na_v_1 channels possess the ankyrin G binding motif, only the nervous system specific cluster of vertebrate sodium channels (Na_v_1.1, Na_v_1.2, Na_v_1.3, Na_v_1.6, and Na_v_1.7) possess a CK2 serine/threonine kinase phosphorylation site downstream of the ankyrin G binding site in the II-III linker (Figures 10, 11B), that greatly increases affinity and required for the formation of stable ankyrin G – sodium channel complexes upon phosphorylation (Xu and Cooper, 2015).

## Ankyring-Specific Na Channel Clustering at Axon Initial Segments and Nodes of Ranvier

The presence of an AnkyrinG-specific Na channel clustering motif correlates with the greater specialization and differentiation of axons from dendrites in chordates, often lacking in invertebrates. Na_v_1 channels in chordates cluster at the proximate ends of axons in AIS, creating fidelity in nerve impulse generation, by ensuring that depolarizing synaptic inputs safely reach threshold necessary for firing action potentials. Another major invention is in structures that increases the speed of nerve propagation. This takes the form of unique invertebrate giant axons (mm in diameter) of the Atlantic squid, for example, which lowers the internal resistance to ionic current flow, and creates a large surface area for sodium channels to populate and contribute to rapid nerve impulses (Hartline and Colman, 2007). An alternative process, and favorable to the limited space for neurons within a cranium and energy efficiency, is myelination, where insulation layers of lipid wrappings of myelin surround axonal membranes. Myelination is omnipresent in craniates, vertebrates which includes all the bony fish and contemporary sharks (gnathostomes) and most basally appearing in a placoderm-like ancestor (Hill et al., 2008). Wraps of insulating myelin, increases conduction speeds by shielding axons from current loss, by increasing transmembrane resistance to current flow, and decreasing the need for input currents to charge the membrane capacitance. Ankyrin-G specific Na_v_1 channel are clustered in periodic bare regions lacking myelination (Nodes of Ranvier), provides a voltage-boost to perpetuate the nerve signal between myelinated internodes lacking Na_v_1 channels (Freeman et al., 2016). Na_v_1 channel localization is inhibited under myelinated regions, and quickly populate along axonal membranes of freshly denervated nerve fibers (England et al., 1990). The close and inverse relationship between the localization of Na_v_1 channels and myelination in axons, relates to an axonal specialization in nervous systems of craniates. The jawless fishes, represented by *Petromyzon marinus*, appear to be the transition group, which rely on squid-like giant axons instead of myelinated axons of the craniates for fast axonal conduction velocities (Teravainen and Rovainen, 1971; Bullock et al., 1984), yet possess the Ankyrin-G specific targeting motif for Na_v_1 channel clustering (Hill et al., 2008) which is lacking in invertebrates.

It should be noted though that myelination isn’t strictly a vertebrate innovation, with some invertebrate lineages possessing myelinated axons (internodes) and bare nodes resembling that of vertebrate axons. Examples of myelinated invertebrate axons are found in crustaceans (malacostraca, including cecapod shrimp and copepods) and annelids (polychaetes and oligochaetes) (Hartline and Colman, 2007). There are also nodal-like clusters of Na_v_1 sodium channels along unmyelinated fibers of the marine snail, *Aplysia californica* (Johnston et al., 1996). Although lacking a vertebrate-like Ankyrin G dependent clustering mechanisms, different invertebrate groups must possess a parallel mechanisms to cluster Na_v_1 channels, given that invertebrates possess clustered sodium channels in unmyelinated axons and vertebrate-like Nodes of Ranvier within myelinated axons. Populations of sodium channels strategically localized along axons is necessary to generate the high fidelity, unidirectional action potentials that are found in both invertebrate and vertebrate nervous systems (Freeman et al., 2016).

## Sodium (Na_v_2, Na_v_1) and Calcium (Ca_v_1, Ca_v_2) Channels Often Possess Recognizable Pdz Binding Motifs at C-Terminal Tails

**FIGURE 12:**
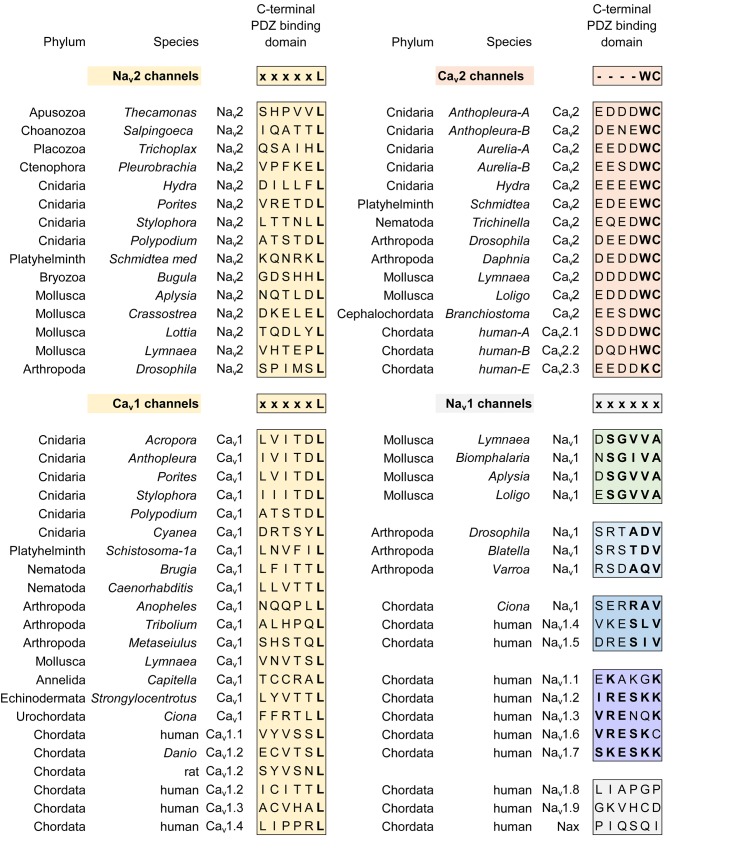
Alignment of amino acid sequences coding for the C-terminal ends of calcium channels (Ca_v_1 and Ca_v_2) and sodium channels (Na_v_2 and Na_v_1) where there are conserved sequence motifs associated with binding to different classes of PDZ domain containing proteins. There is a surprising consistency in a xxxxL motif conserved between Na_v_2 and Ca_v_1 channels (yellow color), both of which constitute the basal complement of high voltage-activated calcium channels and sodium channels present in genomes of single cell choanoflagellates such as *S. rosetta*. The Na_v_1 and Ca_v_2 channels, which are likely derived from Na_v_2 and Ca_v_1 channels, respectively, possess different sets of PDZ binding domain motifs (denoted by orange, green, blue, purple colors). PDZ binding motifs uniquely diverge within the Na_v_1 channel class where molluscan, arthropod, and different human sodium channels associate with separate classes of PDZ domain containing proteins, contributing to their specific localization within their unique cellular environments. The “SLV/SIV” C-terminal PDZ binding motif in mammalian Na_v_1.4 and Na_v_1.5, for example, aids in the complexing of these Na_v_1 channels to syntrophins and SAP97, for the cellular targeting within skeletal and cardiac muscle, respectively. Ca_v_3 channels lack conservation in a C-terminal PDZ domain. Amino acid sequences were aligned using MUSCLE 3.7 (Edgar, 2004) within EMBL-EBI web interface (Chojnacki et al., 2017).

The very ends of the C-termini of particular classes of calcium and sodium channels, like other ion channels or receptors (e.g., Kv channels or NMDA receptors), can possess conserved sequence motifs that are known to associate with different classes of PDZ domains containing proteins, which are known to play supporting roles in the anchoring of ion channels or receptors to proteins associated with the cytoskeleton (Figure 12; Lee and Zheng, 2010; Hui et al., 2013). Na_v_2 channels, including homologs in the earliest branching representatives, apusozoan (*Thecamonas*) and choanoflagellate (*Salpingoeca*) possess a common PDZ binding motif: xxxxL (Figure 12). The xxxxL motif is shared amongst most Ca_v_1 channel homologs too including invertebrate (Ca_v_1) and vertebrate (Ca_v_1.1 to Ca_v_1.4), suggesting that the C-terminal xxxxL motif may have appeared in the first common ancestors of Na_v_2/Ca_v_1 channels in a single cell choanoflagellate-like ancestor (Figure 12). Ca_v_2 channels, evolved as a separate calcium channel class from Ca_v_1 channels to service the presynaptic release of transmitters at nerve synapses, and possess a different C-terminal motif of xxxWC that is shared amongst invertebrate Ca_v_2 and vertebrate Ca_v_2.1 and Ca_v_2.2 channels (Figure 12).

Different Na_v_1 homologs of invertebrate groups and different vertebrate Na_v_1 isoforms, have variable C-terminal PDZ binding motifs than the xxxxL motif common to the Na_v_2 and Ca_v_1 channels. The different PDZ binding motifs likely reflect a difference in PDZ domain containing proteins that supporting the scaffolding of Na_v_1 channels to the cytoskeleton in different invertebrate groups and amongst different vertebrate Na_v_1 isoforms. Arthropod Na_v_1 channels have a common PDZ binding motif of xxA/TDV, while molluscan Na_v_1 channels have a common PDZ binding motif of SGVVA (Figure 12). Vertebrate Na_v_1 channel PDZ binding motifs have a common motif that loosely resembles R/K ESKK in nervous system related sodium channels (Na_v_1.1, Na_v_1.2, Na_v_1.4, Na_v_1.6, and Na_v_1.7) while skeletal muscle specific sodium channel (Na_v_1.4) and cardiac-specific sodium channel (Na_v_1.5) have a PDZ binding motif of R/K ES L/I V (Figure 12). A role for PDZ binding motifs in Na_v_ channels has been illustrated for the last three residues (SIV) of the PDZ domain binding motif in the association of syntrophins and SAP97 to Na_v_1.5 and K_v_ channels. PDZ domain containing scaffolding proteins are required for the protein targeting of Na_v_1.5 and K_v_ channels to the lateral cardiomyocyte membrane specifically, without influencing their location at intercalated disks (Shy et al., 2014). This C-terminal PDZ binding motif also appears to protect Na_v_1.5 channels from proteosomal degradation within cardiomyocytes (Shy et al., 2014). C-terminal PDZ binding motifs are not always conserved in different homologs of Na_v_1, Na_v_2, Ca_v_1, and Ca_v_2 channels and a consensus sequence is notably absent at the C-terminal ends of Ca_v_3 channels. The conservation of C-terminal PDZ binding motifs amongst different calcium and sodium channel classes is suggestive of their importance, but their lack of ubiquity suggest that they are not obligatory factors involved in the regulation of sodium and calcium channels.

An apparently obligatory association for different Na_v_1 sodium channel representatives is the accessory, Na_v_β subunits of Na_v_1 channels. The Na_v_β subunits of Na_v_1 channels are non-homologous in structure between different animal groups, suggestive of differing protein interactions and cellular environments for sodium channel localization in tissues of different animal groups (see Table 1). The differing Na_v_β subunits for Na_v_1 channels in different animal groups is consistent with a similar divergence of C-terminal PDZ binding motifs amongst differing invertebrate Na_v_1 channels (e.g., arthropod versus mollusk) and vertebrate Na_v_1 channels (nervous vs. muscle tissue specific isoforms).

**Table 1:** Comparisons between sodium and calcium channel accessory subunits^#^.

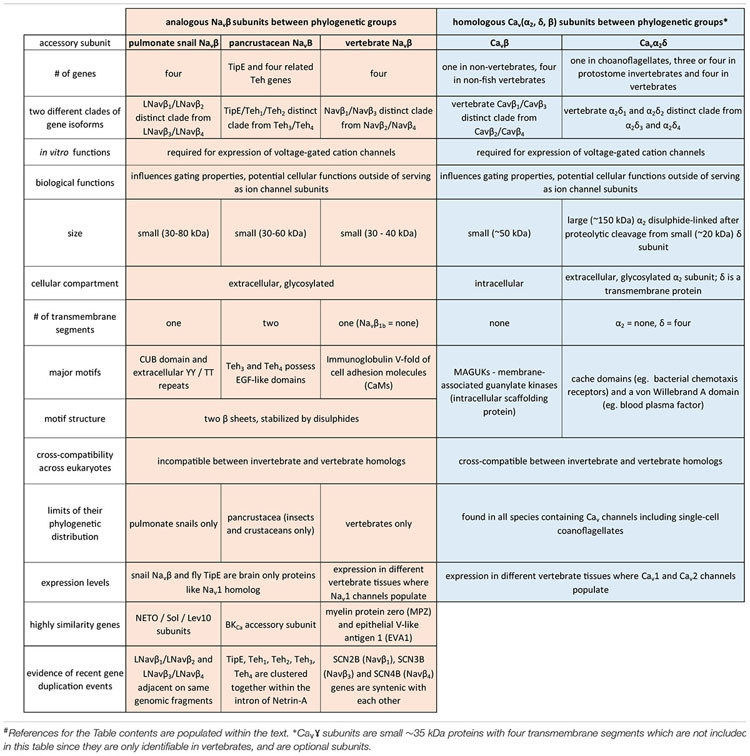

## Auxiliary Ca_v_β Subunits Homologs Exist in All Genomes Containing Ca_v_1 and Ca_v_2 Calcium Channels

Most ion channels are complexed with auxiliary subunits that can serve a chaperoning role to help traffic and target ion channels to specific membrane locales and prevent their proteosomal degradation (Dolphin, 2012; Calhoun and Isom, 2014). Auxiliary subunits also modulate channel gating, such as the kinetic rate of gating or shifts in the voltage-sensitivity of activation or inactivation properties of voltage-gated channels (Dolphin, 2012; Calhoun and Isom, 2014). An important context for assessing the subset of sodium channel specific accessory subunits, is to compare them with the accessory subunits of calcium channels. High voltage-activated Ca_v_1 and Ca_v_2 calcium channels form a multi-protein complex with Ca_v_α_2_δ and Ca_v_β subunit homologs (Dolphin, 2012). The high homology of these accessory subunits is evident in the ability to mix and match the four mammalian Ca_v_β_1_ to Ca_v_β_4_ subunits with singleton Ca_v_β homologs from invertebrate species, like jellyfish (Jeziorski et al., 1999) or snail (Dawson et al., 2014) or squid (Kimura and Kubo, 2003), and these different accessory subunits will alter the biophysical properties or expression features of invertebrate or mammalian Ca_v_1 or Ca_v_2 channels. Both an accessory Ca_v_α_2_δ and Ca_v_β subunit are required for expressing calcium channels *in vitro*, even for the Ca_v_1 homolog from single cell choanoflagellate, *S. rosetta* (Mehta, 2016). The importance of the Ca_v_α_2_δ and Ca_v_β subunit for calcium channels is evident in the ubiquity of these accessory subunit in genomes of known species with calcium channel homologs, including the basal representative in *S. rosetta*, while the Ca_v_α_2_δ and Ca_v_β subunits are not found in apusozoan *Thecamonas trahens* or similar choanoflagellate, *Monosiga brevicollis*, which possess an Na_v_2 but are lacking a calcium channel homolog in their genome (Liebeskind et al., 2011). Ca_v_β subunits possess conserved guanylate kinase (GK), and Src-homology 3 (SH3) domains, which are featured in the related family membrane-associated guanylate kinase (MAGUK) family (Buraei and Yang, 2010). MAGUKs serve as protein scaffolds in the organization of multiprotein complexes at specialized membrane locales such as synapses or other cellular junctions (Zhu et al., 2016).

## Auxiliary Na_v_β Subunits Resemble Cell Adhesion Molecules, but they are Analogous Structures in Different Animal Subphyla

Known sodium channel β subunits (Na_v_β) resemble cell adhesion molecules (CAMs) but are not structural homologs amongst different animal groups, unlike the calcium channel α_2_δ and β, which are homologous, cross-compatible subunits from single cell choanoflagellates to humans. A comparison of features of accessory sodium and calcium subunits is provided in Table 1.

The four vertebrate Na_v_β_1_ to Na_v_β_4_ subunits resemble CAMs of the immunoglobulin (Ig) superfamily with an extracellular Ig V-fold domain (Isom et al., 1995). These Na_v_β subunits play critical roles in nerve outgrowth, the migration of neurites, and trafficking of Na_v_1 channels to specialized positions in nerve membranes (O′Malley and Isom, 2015). CAM domains of Na_v_β_1_ and Na_v_β_2_ subunit isoforms are reported to contact and adhere CAM domains of Na_v_β subunits and with other CAM domain containing proteins within particular cells or between CAM domains linking adjacent cells (Malhotra et al., 2000). Na_v_β facilitates the recruitment of Ankyrin-G to points of cell–cell contact, and tether vertebrate nervous system-specific Na_v_1 channels to AIS and nodes of Ranvier (Chen et al., 2004).

The equivalent to the vertebrate Na_v_β subunits were discovered as TipE (Temperature-induced paralytic E) gene (Hodges et al., 2002) and closely related TipE-homologous genes (TEH1-4) (Derst et al., 2006) in fruit fly (*Drosophila*). Fly mutants of the accessory Na_v_β subunit TipE generates a similar temperature-sensitive paralysis resembling the mutant *para* (short for *paralytic*) phenotype resulting from impaired nerve conduction in fruit flies lacking their functional Na_v_1 channel (*para*) homolog. Double mutants of TipE and *para* genes generates a lethal phenotype in fruit flies (Ganetzky, 1986). Like the vertebrate Na_v_β subunits, co-expression of TipE is necessary to reconstitute the fast channel kinetics of the fruit fly Na_v_1 channel, and greatly enhances the membrane expression of fruit fly Na_v_1 channels *in vitro* (Warmke et al., 1997). TipE and TEH homologs don’t resemble vertebrate Na_v_β subunits and more resemble the structure of accessory subunits to BK (*Slo*, maxi-K, KCa1.1) channels, with two transmembrane domains separated by an extracellular loop containing disulphide bridges (Derst et al., 2006). Extracellular loops in some TEH homologs can resemble CAMs within calcium-binding epidermal growth factor (EGF)-like domains (Derst et al., 2006). Recently, we discovered a family of calcium-binding, CUB domain-contain proteins in *Lymnaea* pond snails, identified as Na_v_β subunits in the protein complex bound to Na_v_1 sodium channels (Fux et al., 2016). Like the vertebrate Na_v_β subunits which can function as CAMs, CUB domain containing proteins have various roles such as in developmental patterning, tissue repair, angiogenesis and cell signaling (Gaboriaud et al., 2011). The most closely related vertebrate CUB domain protein to snail Na_v_β subunits is neuropilin-1 and neuropilin-2, which possess CAMs and serve as co-receptors for class-3 semaphorins, which contribute to axon guidance in the vertebrate nervous system (Nakamura and Goshima, 2002). Other CUB domain containing proteins can be auxiliary subunits, such Sol-1 and Sol-2 that are auxiliary subunits of AMPA glutamate receptors in nematode, *Caenorhabditis elegans* (Wang et al., 2012). Similar Neto1 and Neto2, CUB domain containing proteins, co-assemble with vertebrate glutamate receptors (NMDA and kainite) to modulate their channel gating and enhance their membrane trafficking (Wyeth et al., 2014). The common thread between the Na_v_β subunits is cellular adhesion involving extracellular motifs, but in different structural forms within CUB domains (gastropod snails) (Fux et al., 2016), EGF-like domains (insects) (Derst et al., 2006), or Ig V-fold (vertebrates) (Isom et al., 1995; see Table 1).

## Unique Protein Structures of Na_v_β Subunits Populate the Unique Nervous System Environment Within Different Animal Sub-Phyla

Na_v_β subunit homologs possess a highly restricted distribution within animal sub-phyla. We could identify close homologs to the CUB-domain containing Na_v_β subunits from giant pond snail (*Lymnaea stagnalis*) in a closely related pulmonate freshwater pond snail, *Biomphalaria glabrata*. We could not find Na_v_β subunit homologs outside of pulmonate snails, including the gastropod snail genomes of *Aplysia californica* (California sea hare, marine snail) or *Lottia gigantea* (giant owl limpet), or any non-snail species within the Phylum Mollusca (Fux et al., 2016). Similarly, Tip-E and TEH subunit homologs first identified in fruit flies, are limited to insect and crustacean genomes within the Phylum Arthropoda, and not identifiable, within other arthropods such as chelicerates or myriapods (Li et al., 2011). The vertebrate Na_v_β subunits are similarly restricted in distribution to the subphylum Vertebrata, and absent for example, in genomes of non-vertebrate chordates.

Analogous Na_v_β subunits likely appeared alongside Na_v_1 channels within the first nervous systems, in a common ancestor to extant cnidarians. The more ancestral sodium channel isoform, Na_v_2, likely requires no accessory Na_v_β subunit. Na_v_2 from single cell choanoflagellate, *S. rosetta* expresses well in a vertebrate surrogate cell (such as HEK-293T cell lines or *Xenopus* oocytes) without a co-expressed accessory Na_v_β subunit (Mehta, 2016). Almost all invertebrate Na_v_1 channels do not reach detectable levels for electrophysiological recording using standard *in vitro* expression system in vertebrate cells, outside of insect [fruit fly (Warmke et al., 1997), mosquito (Du et al., 2013), honeybee (Gosselin-Badaroudine et al., 2016), bumblebee (Wu et al., 2017), or cockroach (Du et al., 2009a)] or chelicerate (varroa mite) (Du et al., 2009b). The generally poor expressibility of invertebrate Na_v_1 channels outside their native environment and their requirements for very different structures representing Na_v_β subunits, is consistent with the highly variable composition of invertebrate nervous systems, and the appearance of Na_v_1 channels - protein complexes adapted for the unique neuronal environments within different animal sub-phyla. This is different from calcium channels where all the invertebrate Ca_v_1 (Dawson et al., 2014), Ca_v_2 (Huang et al., 2010) and Ca_v_3 (Senatore and Spafford, 2010) homologs are readily expressible *in vitro*, such as those derived from pond snail, *L. stagnalis*.

## Expansion of Vertebrate Sodium Channel Isoforms Indicate Greater Possibilities in Electrical Signaling as Well as Some Redundancy

Sodium channels rapidly expand in gene numbers from the solitary Na_v_1 channel gene in most invertebrates and non-vertebrate chordates to nine vertebrate genes, Na_v_1.1 to Na_v_1.9 (Alexander et al., 2017). Vertebrate isoforms contribute to the primary action potential generation and conduction in neurons (Na_v_1.1, Na_v_1.2, Na_v_1.3, Na_v_1.6, Na_v_1.7, and Na_v_1.8) or in skeletal or heart muscle, respectively (Na_v_1.4 or Na_v_1.5) (Alexander et al., 2017). Most of the vertebrate channel isoforms (Alexander et al., 2017), (with notable exceptions) generate native currents with characteristics resembling the typical invertebrate (Cole and Moore, 1960; Hille, 1968; Grigoriev et al., 1996; Spafford et al., 1996; Warmke et al., 1997; Mehta, 2016) sodium currents (e.g., midpoint of activation = -30 mV to -20 mV, a rapid inactivation decay (τ = ∼1 ms) and possess a nanomolar drug sensitivity to tetrodotoxin) (Figure 9A and Table 2). Each vertebrate sodium channel gene varies in their pattern of expression during development, and in their tissue and sub-cellular localization (Alexander et al., 2017). Na_v_1.3, for example, specializes as an embryonic and neonatal sodium channel that re-expresses in adults after nerve axotomy or injury (Hains and Waxman, 2007). An L858H sodium channel mutation reported in Na_v_1.7 generates a depolarized resting membrane potential in human patients with erythromelalgia (Rush et al., 2006). Expression of the higher threshold sodium channel (Na_v_1.8) creates a hyper-excitability phenotype in sympathetic neurons and its absence generates a hypo-excitability phenotype in dorsal root ganglion neurons (Rush et al., 2006). The higher threshold Na_v_1.8 channels uniquely drives a greater excitability in the more depolarized membranes of sympathetic neurons in the diseased condition (Rush et al., 2006). The lower threshold sodium channels normally responsible for the action potential upstroke (Na_v_1.1 and Na_v_1.6) are refractory or inactivated due to the more depolarized resting membrane potential of the diseased condition in dorsal root ganglion neurons (Rush et al., 2006). The differing consequences in this example of a single mutation in human Na_v_1.7 illustrate how the cellular phenotype critically depends on the unique properties of the ensemble of sodium channel genes expressed in different neuron types (e.g., sympathetic neurons vs. dorsal root ganglion subtypes).

**Table 2:** Basic characteristics of sample sodium channels including human isoforms.

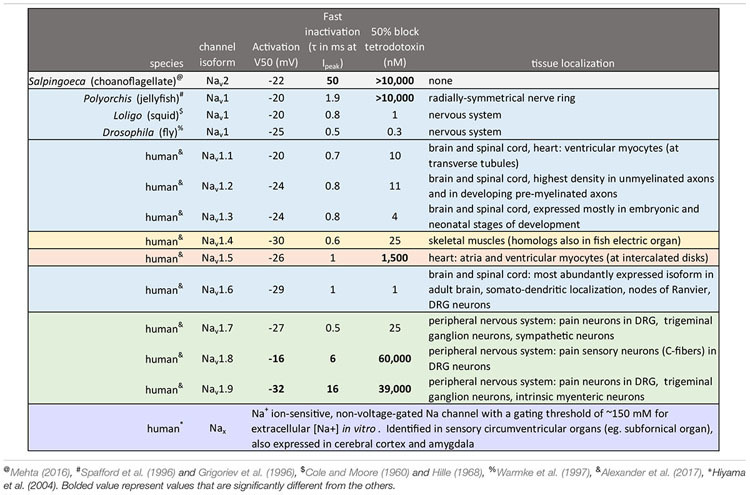

There are examples of apparent redundancies suggesting that the expansion of vertebrate gene isoforms does not always fulfill a requirement for functional diversity. Na_v_1.6 is the dominantly expressed, adult isoform in AIS and nodes of Ranvier (Van Wart and Matthews, 2006). Despite the critical role that it plays, Na_v_1.6 is remarkably well-compensated by Na_v_1.2 channels expressed in neocortical pyramidal neurons in response to the tissue-selective knockdown of Na_v_1.6 (Katz et al., 2018). The only difference in Na_v_1.6-null compared to wild type neocortical neurons is a reduction in the size of the persistent sodium current (Katz et al., 2018).

## “Persistent” Sodium Currents are Common in Different Invertebrates and Vertebrates, While Observed “Resurgent” Sodium Currents are Likely an Invention Within Vertebrates

“Persistent” or “late” sodium currents are residual (<5%), steady-state, sub-threshold sodium currents that are often generated from the incomplete inactivation of Na_v_1 channels after the fast transient sodium spike, or as a trickle of “window current” of open and non-inactivated Na_v_1 channels at rest (Crill, 1996). Persistent sodium currents can drive pace-making (Bevan and Wilson, 1999), burst firing (Azouz et al., 1996) or amplify excitatory post-synaptic potentials (Deisz et al., 1991). Persistent sodium currents have been identified across neurons of every major phyla (invertebrate and vertebrate) (Kiss, 2008) and vertebrate cardiac muscle (Moreno and Clancy, 2012) (see Figure 9B). Persistent sodium currents are readily generated with as little as a single amino acid change, presence of a gating modifying toxin or after co-expression of a Na_v_β subunit isoform in vertebrates which can influence the voltage-dependence or kinetics of inactivation of Na_v_1 channels (Qu et al., 2001; Aman et al., 2009; Savio-Galimberti et al., 2012).

A striking example of the independent evolution of persistent sodium currents is in the specialized Na_v_1.4 channel isoforms for the muscle-derived electric organs of weakly-electric fish, which can generate electrical pulses in excesses of 1 kHz for sensing their nocturnal environment and the electro-communication with other fish (Thompson et al., 2018). The electromotor neurons of weakly-electric fish can fire at a faster rate of any known animal neuron, using a persistent sodium current generated from the modification of its structural elements for inactivation (S4-S5 linker of Domain IV) of their Na_v_1.4 channels (Thompson et al., 2018).

A different type of inter-spike sodium current contributing to pace-making is the “resurgent” sodium current (Figure 9C) (Raman and Bean, 1997). The resurgent sodium current is likely an adaptation limited to vertebrates, reported for particular neuronal cell types including the cerebellum and brainstem, globus pallidus, the hippocampus and dorsal root ganglia (Lewis and Raman, 2014). The “resurgent” current flows through the same channels which generate the “transient” and “persistent” components of the Na_v_1 channel currents (Lewis and Raman, 2014; Figure 9C). The “resurgent” sodium current is characterized by a rise, peak and subsequent fall of sodium current during the repolarization phase of the action potential, after the action potential overshoot and repolarization of the membrane below zero mV (Figure 9C) (Raman and Bean, 1997). A fraction of the Na_v_1 channel pores are considered to be under an “open channel” blockade during membrane depolarization, with a visible “resurgent” current appearing at the rate of unblocking of channel pores by the “blocking particle” upon action potential repolarization, with a surge of inward current enhanced by the greater driving force for ion flow through Na_v_1 channels at the more hyperpolarized membrane potentials during repolarization (Lewis and Raman, 2014). One of the likely contributors as the “blocking particle” is the unique C-terminal insert in the vertebrate Na_v_β_4_ subunit which contains positively charged and hydrophobic residues that can reconstitute resurgent currents in neurons that do not exhibit an endogenous resurgent current (Grieco et al., 2002). Not all neurons containing resurgent sodium currents express Na_v_β_4_ subunit and co-expression of Na_v_β_4_ subunit alone does not reconstitute the resurgent sodium current *in vitro* (Lewis and Raman, 2014). There are likely other mechanisms alongside Na_v_β_4_ subunits which contribute to generating resurgent sodium currents in vertebrate neurons. The regulation by Na_v_β_4_ is reminiscent of the blockade of the inward-rectifying potassium channels by endogenous polyamines, namely spermine, as well as magnesium ions that plug the channel pore at positive potentials (Baronas and Kurata, 2014). It also has similarities in the adaptations of different accessory β subunits which mimic the positively charged and hydrophobic N-terminal “ball” of the “ball-and-chain”, contributing to the fast N-type inactivation in different voltage-gated potassium channels (Bentrop et al., 2001).

## The Origins of the Sodium and Calcium Channels are Contained in Single Cell Eukaryotes

Teasing apart the different ion channel contributions in the electrical signaling of vertebrate tissues has relied on parallel approaches in analyzing expressed genes in surrogate expression systems (e.g., mammalian cell lines or *Xenopus* oocytes) (Tapper et al., 2003). While convenient, *in vitro* expression studies are usually insufficient in recapitulating the condition of the native cell such as the many gene splicing variants expressed natively in cells, complex of associated, accessory subunits, post-translational regulation, and other myriad of critical interactions required for ion channel subunits within specific subcellular locales of different tissues. Surrogate expression systems also can possess endogenous, contaminating sodium currents (He and Soderlund, 2010; Terhag et al., 2010). A parallel approach is to examine a simple eukaryotic model to better understand the functions of voltage-gated sodium and calcium channels, where there are limitations in gene complexity, such as in the choanoflagellates where there are only five gene homologs (Ca_v_1, Ca_v_α_2_δ, Ca_v_β, Ca_v_3, and Na_v_2). From an evaluation of their structural and functional characteristics, it is evident that these five choanoflagellates genes are homologs of the 28 equivalent human sodium and calcium channel genes, packaged in a living organism consisting of a single eukaryotic cell.

## Conclusion and Future Outlook in Studies of Basal Organisms

**FIGURE 13:**
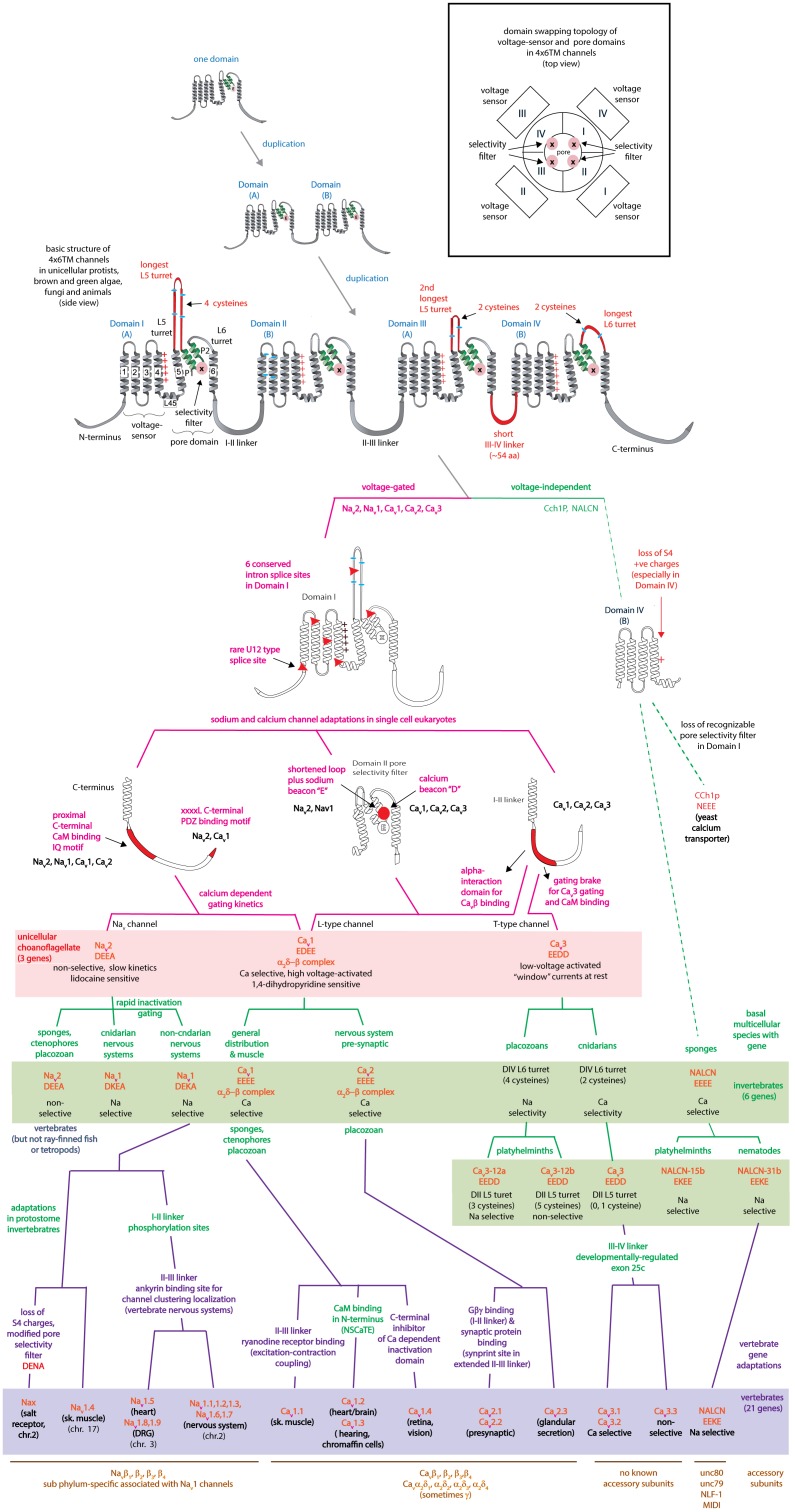
Flow chart illustrating progression of features evolving within 4 × 6 TM cation channels, including the three primary voltage-gated channels contained in single cell choanoflagellates (Na_v_2, Ca_v_1, and Ca_v_3) (pink color, 3 genes) and their diversification and specialization within protostome invertebrates (green color, 6 genes) and vertebrates (purple color, 21 genes).

The groundwork for the appearance of nervous systems and Na_v_1 channels is evident in the first multicellular organisms. Sponge and *Trichoplax* have no traditional nerve structures such as neurons or synapses, but do possess the building blocks for nervous systems including protein homologs for synaptic and scaffolding elements, and cell-cell communication elements that involves many of the classical ionic channels and neurotransmitter receptors of higher organisms (Moroz and Kohn, 2016). The coelenterates were considered a grouping of basal, radial symmetrical animals that superficially resembled each other with rudimentary diffuse nervous nets serving as nervous systems, including the cnidarians (hydra, corals, sea anemones, jellyfish, sea pens) and the ctenophores (comb jellies) (Zapata et al., 2015). Delving into genome comparisons between cnidarians and ctenophores has reveal that the genomic blueprints are starkly different, where ctenophores appear to have an early and parallel evolution to cnidarians and other bilateral animals (Moroz et al., 2014), and lack many of the classical neural elements, including major voltage-gated cation channel classes, including the classical Na_v_1 channels required for fast sodium channel spikes along nerve axons, and Ca_v_2 and Ca_v_3 calcium channels involved in mediating neurotransmitter secretion across nerve synapses or required for pace-making of action potentials, respectively. Single cell choanoflagellates, while more basal to the multicellular ctenophores, possess a complement of single homologs of the major sodium (Na_v_2) and calcium channel subunits (Ca_v_1 and Ca_v_3), plus contain accessory calcium channel subunits (α2δ, Ca_v_β) (Mehta, 2016). It is in the single cell choanoflagellates that we are likely able to resolve the structural and functional basis of which lead to the specialization of electrical signaling in voltage-gated cation channels (see summary flow chart, Figure 13). Species like *S. rosetta* contain both solitary, highly mobile life stages as well as colonial, and more sessile life stages (Dayel et al., 2011), where we can evaluate the fundamental underpinnings for the first appearances of 4 × 6 TM, voltage-gated sodium and calcium channels, which we mostly attribute to specialized roles in the rapid electrical and chemical signaling within multicellular organisms.

## Author Contributions

JF, AM, and JM carried out the experiments for the work that this review is based on. JS wrote the text and made the figures for the manuscript.

## Conflict of Interest Statement

The authors declare that the research was conducted in the absence of any commercial or financial relationships that could be construed as a potential conflict of interest.
